# Measuring protective efficacy and quantifying the impact of drug resistance: A novel malaria chemoprevention trial design and methodology

**DOI:** 10.1371/journal.pmed.1004376

**Published:** 2024-05-09

**Authors:** Andria Mousa, Gina Cuomo-Dannenburg, Hayley A. Thompson, R. Matthew Chico, Khalid B. Beshir, Colin J. Sutherland, David Schellenberg, Roly Gosling, Michael Alifrangis, Emma Filtenborg Hocke, Helle Hansson, Ana Chopo-Pizarro, Wilfred F. Mbacham, Innocent M. Ali, Mike Chaponda, Cally Roper, Lucy C. Okell

**Affiliations:** 1 Faculty of Infectious and Tropical Diseases, London School of Hygiene and Tropical Medicine, London, United Kingdom; 2 MRC Centre for Global Infectious Disease Analysis, Department of Infectious Disease Epidemiology, Imperial College London, London, United Kingdom; 3 Malaria and Neglected Tropical Diseases, PATH, Seattle, Washington, United States of America; 4 Malaria Elimination Initiative, Institute of Global Health, University of California, San Francisco, California, United States of America; 5 Center for Medical Parasitology, Department of Immunology and Microbiology, University of Copenhagen, Copenhagen, Denmark; 6 Department of Infectious Diseases, Copenhagen University Hospital, Copenhagen, Denmark; 7 The Biotechnology Centre, University of Yaoundé, Yaoundé, Cameroon; 8 The Fobang Institutes for Innovation in Science and Technology, Yaoundé, Cameroon; 9 The Faculty of Northwest University, Faculty of Natural and Agricultural Sciences, Potchefstroom, South Africa; 10 Department of Biochemistry, Faculty of Science, University of Dschang, Dschang, Cameroon; 11 Department of Clinical Sciences, Tropical Diseases Research Centre, Ndola, Zambia; University of Glasgow, UNITED KINGDOM

## Abstract

**Background:**

Recently revised WHO guidelines on malaria chemoprevention have opened the door to more tailored implementation. Countries face choices on whether to replace old drugs, target additional age groups, and adapt delivery schedules according to local drug resistance levels and malaria transmission patterns. Regular routine assessment of protective efficacy of chemoprevention is key. Here, we apply a novel modelling approach to aid the design and analysis of chemoprevention trials and generate measures of protection that can be applied across a range of transmission settings.

**Methods and findings:**

We developed a model of genotype-specific drug protection, which accounts for underlying risk of infection and circulating genotypes. Using a Bayesian framework, we fitted the model to multiple simulated scenarios to explore variations in study design, setting, and participant characteristics.

We find that a placebo or control group with no drug protection is valuable but not always feasible. An alternative approach is a single-arm trial with an extended follow-up (>42 days), which allows measurement of the underlying infection risk after drug protection wanes, as long as transmission is relatively constant. We show that the currently recommended 28-day follow-up in a single-arm trial results in low precision of estimated 30-day chemoprevention efficacy and low power in determining genotype differences of 12 days in the duration of protection (power = 1.4%). Extending follow-up to 42 days increased precision and power (71.5%) in settings with constant transmission over this time period. However, in settings of unstable transmission, protective efficacy in a single-arm trial was overestimated by 24.3% if recruitment occurred during increasing transmission and underestimated by 15.8% when recruitment occurred during declining transmission. Protective efficacy was estimated with greater precision in high transmission settings, and power to detect differences by resistance genotype was lower in scenarios where the resistant genotype was either rare or too common.

**Conclusions:**

These findings have important implications for the current guidelines on chemoprevention efficacy studies and will be valuable for informing where these studies should be optimally placed. The results underscore the need for a comparator group in seasonal settings and provide evidence that the extension of follow-up in single-arm trials improves the accuracy of measures of protective efficacy in settings with more stable transmission. Extension of follow-up may pose logistical challenges to trial feasibility and associated costs. However, these studies may not need to be repeated multiple times, as the estimates of drug protection against different genotypes can be applied to different settings by adjusting for transmission intensity and frequency of resistance.

## Abstract 2

Using a novel modelling approach, Mousa and colleagues investigate the design and analysis of malaria chemoprevention trials and generate measures of protection that can be applied across a range of transmission settings.

## Author summary

**Why was this study done?:**

- In 2022, the World Health Organisation (WHO) released updated guidelines for the implementation of malaria chemoprevention and guidance for undertaking chemoprevention efficacy studies.
- We sought to understand how study design and trial setting characteristics influence the ability to assess chemoprevention efficacy and duration of drug protection.
- The impact of drug resistance on chemoprevention is not well understood, and there is a need for a method that produces measures of drug resistance effects, which can be applied to different settings.

**What did the researchers do and find?:**

- We fitted a Bayesian Markov Chain Monte Carlo (MCMC) to simulated trial data for a number of trial designs and scenarios.
- We find that the WHO recommendation of 28 days of follow-up is insufficient to precisely estimate duration of drug protection or protective efficacy.
- In seasonal settings, in the absence of a control arm, it is impossible to distinguish between changing transmission and waning drug protection when estimating protective efficacy.

**What do these findings mean?:**

- Where feasible, particularly in settings of seasonal transmission and/or trials without a control arm, extending follow-up to 42 or 63 days can substantially improve the ability to accurately estimate chemoprevention outcomes.
- Although long follow-up can improve precision and power, decisions on study design should also consider costs and trial feasibility.

## Introduction

Chemopreventive treatment of vulnerable groups is an essential component of malaria control. Its aim is to clear existing asymptomatic infections and provide ongoing protection against new infections and clinical disease [1]. Chemoprevention interventions include intermittent preventative treatment in pregnancy (IPTp), perennial malaria chemoprevention (PMC), seasonal malaria chemoprevention (SMC) in children, intermittent preventive treatment in school-aged children (IPTsc), post-discharge malaria chemoprevention (PDMC), mass drug administration (MDA), or reactive focal MDA (rfMDA) [1]. In children, SMC and PMC involve the administration of sulfadoxine-pyrimethamine (SP) alone or in combination with other antimalarials, such as amodiaquine (SP-AQ).

Until recently, SMC was limited to the Sahel region, as the initial recommendation was restricted to areas of Africa with highly endemic, very seasonal malaria transmission and high SP-drug susceptibility [2]. Similarly, the initial recommendation of PMC (previously called IPTi) stated that the intervention should only be given in areas where resistance markers were below a specified threshold [3]. In June 2022, the World Health Organisation (WHO) published new, less prescriptive chemoprevention guidelines that encourage chemoprevention programmes to consider a wider variety of drugs and delivery in new geographies, with locally tailored strategies [1].

The WHO Global Malaria Programme published guidelines for the design and analysis of chemoprevention efficacy studies (CPES) [4]. In their standardised approach, chemoprevention failure is defined as asexual parasitaemia by microscopy within 28 days post-dosing. This approach helps harmonise surveillance across countries, but the main challenge is that findings will be context-specific, varying with underlying transmission intensity and circulating resistance genotypes. In programmes where SP-based chemoprevention is deployed, a priority is to understand how parasite resistance to SP might impact protection, particularly in parts of Eastern and Southern Africa where resistance-associated mutations in the *dhps* and *dhfr* genes are common [5].

Ethical considerations preclude the inclusion of a control group in settings where chemoprevention is the standard of care. Without a control group, it is difficult to distinguish the effect of drug protection from the effect of the local level of malaria incidence. Current guidelines on sample size calculations [4] propose predicting the proportion infected based on microscopy prevalence on the day of treatment (day 0) and the relationship between prevalence and incidence estimated from previous studies [6]. However, in previous analyses, EIR estimates greatly deviated from those predicted from prevalence data [7]. Therefore, when analysing a study, estimating infection rates during the trial may produce more accurate results on protective efficacy.

A second challenge associated with the current WHO-proposed guidelines for chemoprevention trials is that, although they include the reporting of resistance-associated mutation prevalence, there are no guidelines on incorporating resistance genotypes in the analysis to estimate their impact on protective efficacy. Certain parasite mutations are strongly associated with treatment failure in symptomatic patients and may also reduce the duration of chemoprevention (i.e., resistant parasites can reinfect earlier after chemoprevention) [7–9], although, to date, this has been largely uncharacterised [10].

Previous studies have quantified the interval of protection against malaria infection by analysing reinfection data from randomised controlled treatment or chemoprevention trials of lumefantrine, piperaquine, and amodiaquine (the long-acting partner drugs of artemisinin-combination therapies (ACTs)) [7,11] as well as SP-AQ [12]. In these models, the probability of a new infection is a function of both the transmission level in a given area and drug protection. Estimation of the infection rate in a particular setting is informed by data either from a control group or from treated cohorts as the drug protection wanes.

Here, we extend previous modelling approaches to explore considerations and challenges in chemoprevention study design by developing a novel method to quantify protective efficacy, which can be used for the design and analysis of future malaria chemoprevention trials. To the best of our knowledge, this is the first method that produces estimates of protection that can be transposed to other settings with different transmission and frequency of resistance, enabling estimation of chemoprevention efficacy in areas where these trials are not conducted. In our modelling approach, we consider participant characteristics, transmission intensity, seasonality, drug resistance frequency, genotype-specific efficacy estimation, and study-design characteristics, i.e., presence/absence of a control arm and length of follow-up.

## Methods

### Deterministic model

In a chemoprevention trial, asymptomatic individuals are followed and assessed for the presence of a new infection at different time-points. In the absence of chemoprevention, the incidence of new infections (*Λ*) at time *t* is assumed to be constant in perennial settings.

In individuals who have taken chemoprevention, we model the incidence of infection, *Λ*_*C*_, at time *t* after receiving chemoprevention as:

$$\Lambda_{C}(t)=\Lambda (1-e^{{-(\frac{t}{\lambda })}^{w}}),$$

where $e^{{-(\frac{t}{\lambda })}^{w}}$ is the probability of being protected by chemoprevention and is assumed to follow a Weibull survival curve with scale parameter *λ* and shape parameter *w*. The mean duration of protection from a new infection is determined as:

$$Mean\;duration\;of\;protection=\lambda \Gamma \left(1+\frac{1}{w}\right),$$

where *Γ* denotes the gamma function.

### Modelling effects of resistance

The probability of being successfully infected with a resistant parasite is dependent on the frequency of resistance in the parasite population, F_R_, as well as genotype-specific drug protection. We extended the above model to include the possibility of infection with resistant and sensitive parasite genotypes, with durations of protection specific to each genotype or strain (here used interchangeably). We assumed that the duration of drug protection will be different against resistant (R) and sensitive (S) strains (*λ*_*R*_, *λ*_*S*_, and *w*_*R*_, *w*_*S*_). The model assumes that only 1 type of parasite strain can be acquired during each half-daily time step. We also assume some PCR failures in the lab, resulting in missing data on genotypes.

The deterministic equations describing the cumulative proportions infected with resistant and sensitive strains during a trial can be found in in S1 File. The model also has the facility to include more than 2 strains and to account for mixed infections. We ran model simulations in R version 4.2.2 as a discrete-time model (simulated data and code can be accessed at https://github.com/AndriaMousa/chemoprevention-trial-code.git).

### Simulations

To understand how different epidemiological settings and study designs affect the estimation of chemoprevention efficacy, we generated stochastic simulations of a large number of trials with 1 or 2 strains. A single-strain model can be used to quantify effects related to the overall chemoprevention impact, and a multistrain model to quantify genotype-differences. For a given sample size and scenario, we simulated multiple datasets using the stochastic model and then fitted the deterministic model to calculate precision and power. For the 2-strain model, we estimate the power to detect a significant difference in the mean duration of protection between 2 strains. The assumptions used to generate the simulated data are detailed in Table 1 and S1 File. The conditions underlying the baseline scenario, against which all scenarios were compared, are also shown in Tables 2 and 3. We explored variations in the incidence/prevalence of infection, seasonality, loss to follow-up, length of follow-up, sample size, and the presence or absence of a control group.

**Table 1:** Model parameters used in the simulations. Definitions and values used in the main analysis and sensitivity analyses.

Parameter name (symbol)	Units	Main analysis value	Sensitivity analysis values	Source(s)
force of infection (Λ)	infections per person per year	10 ippy*	5 ippy	-
Parasite prevalence	percentage	40%	30%	-
Follow-up	days	63 days	28 days, 42 days	[4,13]
Loss to follow-up	percentage	10%	20%	[14]
Mean duration of protection against any^†^ parasite (μ)	days	20 days	15 days, 25 days	[7,11,12]
Weibull scale parameter of protection against any parasite (λ)	-	$\frac{\mu }{\Gamma (1+\frac{1}{w})}$	-	-
Weibull shape parameter of protection against any parasite (w)	-	5	-	[7,11,12]
Frequency of resistant parasite (*F*_*R*_)	percentage	50%	20%, 90%	-
Frequency of resistant parasite (1−*F*_*R*_)	percentage	50%	80%, 10%	-
Mean duration of protection against resistant parasites (*μ*_*R*_)	days	18 days	15 days, 20 days	-
Weibull scale parameter of protection against resistant parasites (*λ*_*R*_)	-	$\frac{\mu_{R}}{\Gamma (1+\frac{1}{w_{R}})}$	-	-
Weibull shape parameter of protection against resistant parasites (*w*_*R*_)	-	5	-	[7,11,12]
Mean duration of protection against sensitive parasites (*μ*_*S*_)	days	30 days	-	-
Weibull scale parameter of protection against sensitive parasites (*λ*_*S*_)	-	$\frac{\mu_{S}}{\Gamma (1+\frac{1}{w_{S}})}$	-	-
Weibull shape parameter of protection against sensitive parasites (*w*_*S*_)	-	5	-	[7,11,12]
Probability of drug protection against any parasite (δ) at time *t*	probability	$e^{{-(\frac{t}{\lambda })}^{w}}$	-	-
Probability of drug protection against resistant parasites (*δ*_*R*_) at time *t*	probability	$e^{{-(\frac{t}{\lambda_{R}})}^{w_{R}}}$	-	-
Probability of drug protection against sensitive parasites (*δ*_*S*_) at time *t*	probability	$e^{{-(\frac{t}{\lambda_{S}})}^{w_{S}}}$	-	-
Probability of successfully determining infection genotype (*p*_*determ*_)	probability	0.90	0.70	-

*ippy, infections per person per year.

^†^Any parasite refers to a parasite of any genotype and is relevant to the single-strain model.

**Table 2:** Scenarios and parameter inputs for the single-strain model. The 30-day protective efficacy is the deterministic value based on the input parameters shown in the table. For additional scenarios, see Table A in S3 File. Entries in bold denote deviations in the assumptions compared to the baseline scenario.

Varying parameter	Sample size (N)	Length of follow-up (days)	Mean Incidence (ippy*)	Seasonality^¥^	Slide Prevalence (%)	Loss to follow-up (%)	Mean duration of protection (days)	30-day protective efficacy (%)
**Baseline scenario**	**600**	**63**	**10**	**none**	**40**	**10**	**20**	**56.4**
**Variations in transmission**								
↓Incidence	600	63	**5**	none	40	10	20	61.1
↓Incidence and ↓prevalence	600	63	**5**	none	**30**	10	20	61.1
Seasonal setting (increasing transmission)	600	63	10	**start**	40	10	20	52.0
Seasonal setting (decreasing transmission)	600	63	10	**end**	40	10	20	58.3
**Variations in study design**								
↓Sample size	**400**	63	10	none	40	10	20	56.4
Addition of short-acting clearance drug group	**600+200**	63	10	none	40	10	20	56.4
↓Length of follow-up	600	**42**	10	none	40	10	20	56.4
↓↓Length of follow-up	600	**28**	10	none	40	10	20	56.4
Addition of short-acting clearance drug group ↓Length of follow-up	**600+200**	**42**	10	none	40	10	20	56.4
Addition of short-acting clearance drug group ↓↓Length of follow-up	**600+200**	**28**	10	none	40	10	20	56.4

*ippy, infections per person per year.

^¥^No seasonality represents a scenario of perennial transmission. Entries “start” and “end” represent scenarios where participants are recruited during the start and end of the transmission season, respectively (see Methods for details).

**Table 3:** Scenarios and parameter inputs for the 2-strain model. The power to detect a significant difference in the mean duration of protection (% of simulations that reject the null hypothesis of no difference) is shown in the table; R = resistant strain, S = sensitive strain. For additional scenarios, see Table A in S4 File. Entries in bold denote deviations in the assumptions compared to the baseline scenario.

Varying parameter	Sample size (N)	Length of follow-up (days)	Mean Incidence (ippy*)	Slide Prevalence (%)	Freq of R (%)	Infections determined as R or S (%)	Loss to follow-up (%)	Mean duration of protection against S (days)	Mean duration of protection against R (days)	Effect size (days)	Power (%)
**Baseline scenario**	**600**	**63**	**10**	**40**	**50**	**90**	**10**	**30**	**18**	**12**	93.5
**Variations in transmission and study design**											
↓Incidence	600	63	**5**	40	50	90	10	30	18	12	73.3
↓Sample size	**400**	63	10	40	50	90	10	30	18	12	80.1
↓Length of follow-up	600	**42**	10	40	50	90	10	30	18	12	71.5
↓↓Length of follow-up	600	**28**	10	40	50	90	10	30	18	12	1.4
Addition of short-acting clearance drug group ↓Sample size for chemoprevention	**400+200**	63	10	40	50	90	10	30	18	12	93.2
Addition of short-acting clearance drug group ↓Sample size for chemoprevention ↓Length of follow-up	**400+200**	**42**	10	40	50	90	10	30	18	12	90.4
Addition of short-acting clearance drug group ↓Sample size for chemoprevention ↓↓Length of follow-up	**400+200**	**28**	10	40	50	90	10	30	18	12	86.6
**Variations in frequency of resistance**											
↓Freq of R	600	63	10	40	**20**	90	10	30	18	12	69.4
↑Freq of R	600	63	10	40	**90**	90	10	30	18	12	53.4
**Variations in effect size**											
↑effect size	600	63	10	40	50	90	10	30	**15**	15	98.1
↓effect size	600	63	10	40	50	90	10	30	**20**	10	83.0

*ippy, infections per person per year.

### Statistical analysis

Each simulated dataset was analysed to obtain the chemoprevention efficacy and duration of drug protection that would be estimated from that particular simulated trial. The duration of protection was assumed to be different against different genotypic strains and was estimated using Bayesian Markov Chain Monte Carlo (MCMC) models with a random walk Metropolis–Hastings algorithm in Stan [15] using rstan [16]. The time-step used in the model (dt) was 0.5 days. We used relatively uninformative priors for all parameters, and no constraints were imposed on parameter values. We fitted the MCMC model to each simulated trial with 2,500 burnin iterations and 5,000 sampling iterations for each of 4 chains. Convergence of all model fits was assessed visually from the posterior distributions and traceplots, and using a threshold of <1.05 for the Gelman–Rubin’s convergence diagnostic ($\hat{R}$) [17].

The underlying incidence of infection, and the Weibull drug-protection parameters (*λ* and *w)* were estimated. For the 2-strain model, the frequency of the resistant parasite in the population (*F*_*R*_), and the shape and scale parameters for the Weibull survival curve of protection against resistant and sensitive strains (*λ*_*R*_, *λ*_*S*_, *w*_*R*_, *w*_*S*_) were estimated in addition to the incidence. The 30-day efficacy (overall and by strain) was estimated as the percentage of new infections prevented by the drug compared to no chemoprevention. This is different to the metric typically used in SMC trials, which compare clinical incidence over the whole follow-up. For each simulated dataset, we also calculated the 95% credible intervals (CrI) for the median 30-day protective efficacy using the posterior distribution of estimated parameter values across all MCMC iterations. The median width of these 95% CrIs across 1,000 simulations was used as a measure of precision, with wider intervals indicating lower precision (single-strain model; Table 2). For the 2-strain model, power was estimated as the proportion of simulations that reject the null hypothesis, i.e., no significant difference in mean duration of protection between resistant and sensitive strains (Table 2). An alternative power calculation approach using Cox proportional-hazards is also presented.

## Results

### Single-strain model: Transmission intensity

**Fig 1:**
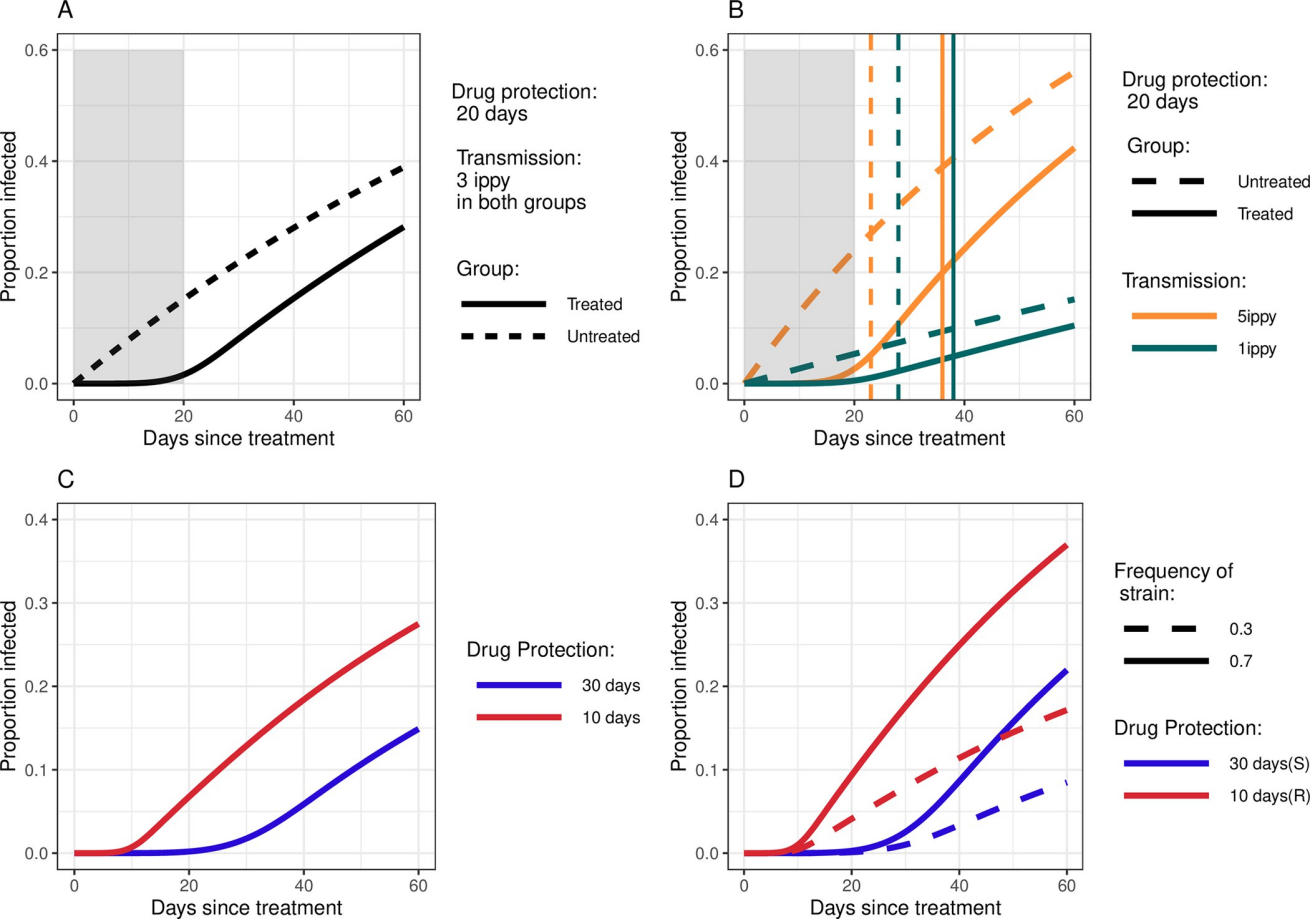
Illustrations showing the proportion infected during follow-up in different scenarios. (**A**) Proportion infected in an untreated cohort and in a cohort treated with a drug that provides a mean of 20 days protection against a new infection. (**B**) This figure shows the equivalent proportions expected in a low transmission setting of 1 infection per person per year (ippy), compared to 5 ippy. Vertical lines denote the median time to a new infection, which varies by both transmission intensity and treatment arm (unlike the fixed duration of protection of 20 days, indicated by the shaded area in panels **A** and **B**) (**C**) Illustration showing the proportion infected in cohorts treated by drugs of varying durations of protection. (**D**) Illustration showing the proportion infected in those living in settings with different frequencies of resistant parasite strains (30% vs. 70%). In this case, we assume that a drug provides 10 days protection against the resistant strain and 30 days protection against the sensitive strain. The infection rates in (**C**) and (**D**) are both set to 5 ippy.

Time to new infection following a drug dose is not equivalent to the duration of protection the drug provides. In a high transmission setting, children become infected quicker compared to a lower transmission setting, for the same interval of drug protection (Fig 1A and 1B).

**Fig 2:**
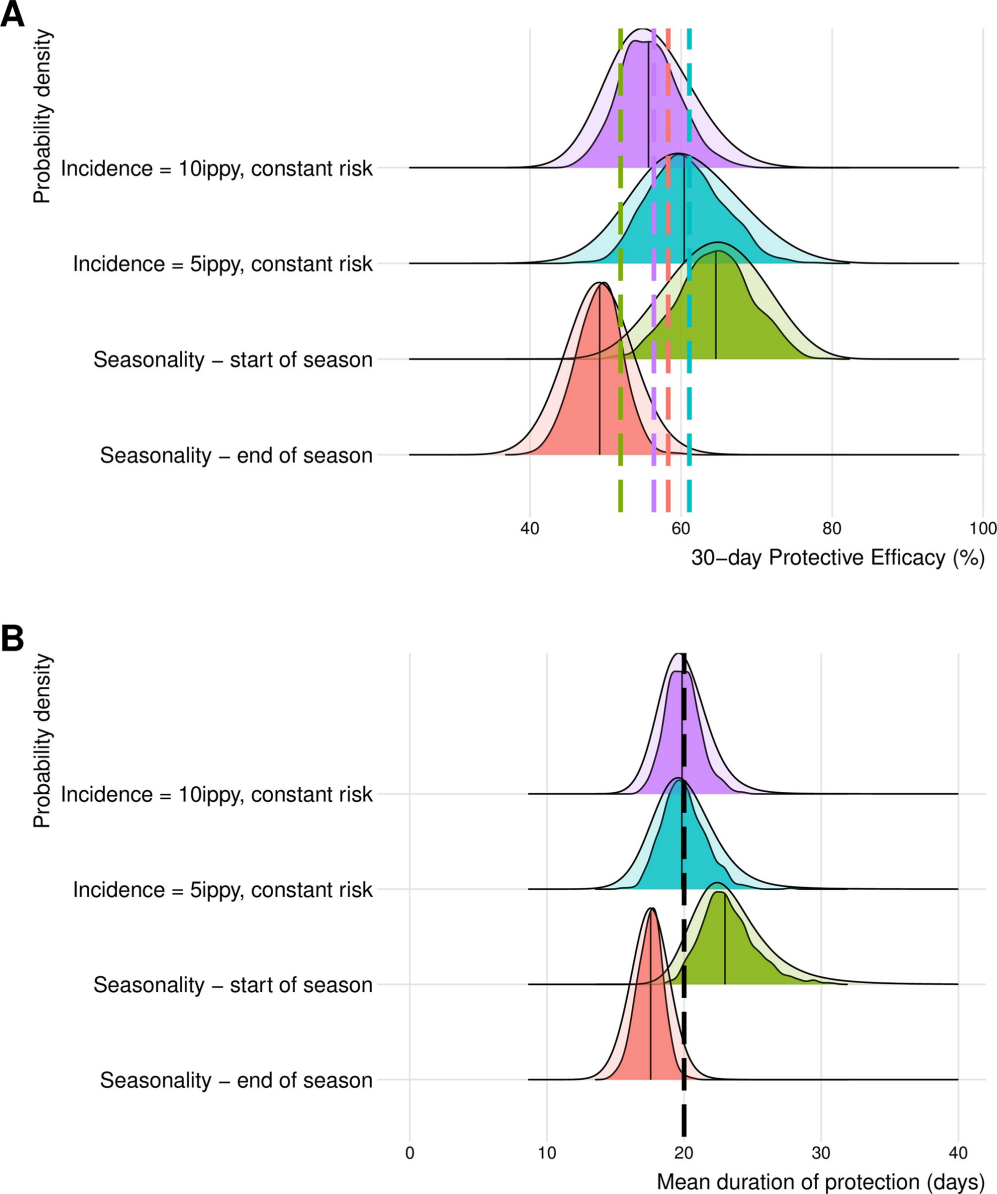
Impact of transmission (level and seasonality/fluctuations) on uncertainty and precision. The probability density distribution of estimated (**A**) 30-day protective efficacy and (**B**) mean duration of protection. All scenarios presented here are a single-arm trial of 600 participants with a true duration of protection of 20 days. Protective efficacy over 30 days is calculated using the estimated posterior parameters as 1 minus the probability of being infected in the treatment arm divided by the probability of being infected in a theoretical control arm over 30 days. Mean duration of protection is also calculated using the estimated posterior parameters. The opaque probability density functions indicate the distribution of all posterior values across all 1,000 simulations (10,000 for each simulation). The darker coloured probability density functions indicate the distribution of the medians across all simulations. Solid vertical black lines show the median values of the estimated distributions. Vertical dashed lines in (**A**) indicate the true deterministic values for the expected 30-day efficacy in seasonal settings and the vertical dashed line in (**B**) indicate the true value for the duration of protection (20 days). ippy, infections per person per year.

WHO recommends chemoprevention in moderate-to-high transmission settings (≥0.25 clinical episodes ppy/parasite rate ≥10%)[1], and all scenarios considered are within that range (see S2 File for relationships between prevalence, infection incidence, and clinical incidence). We used a single-strain model to explore the effect of transmission intensity on estimating chemoprevention efficacy. In a setting of 5 infections per person per year (ippy) 30-day protective efficacy was estimated with lower precision compared to the baseline higher transmission scenario (10 ippy), due to fewer infection events (Fig 2). Across all simulations, the median width of CrIs of protective efficacy was 19.9% (IQR = 18.0% to 21.7%) for the lower transmission setting and 15.2% (IQR = 13.8% to 16.6%) in the scenario of 10 ippy. The respective median widths of CrIs in the mean duration of protection were 6.5 days (IQR = 5.6 to 7.9 days) and 4.8 days (IQR = 4.2 to 5.7 days).

In some scenarios, the incidence of infection may change over time due to seasonality, malaria control interventions, or random fluctuations in transmission intensity. If there is higher incidence at drug receipt when drug protection is high, and incidence declines as the drug protection is waning, this will increase the proportion of cases prevented in the chemoprevention arm, and, hence, increase the true protective efficacy (Table 2). In a study design without a control group, this change in incidence would be unknown. We simulated scenarios where infection risk either increases or decreases over time and fit the model assuming a constant infection risk (S1 File). The lack of knowledge of the changing transmission resulted in an overestimation of 30-day protective efficacy when participants were recruited at the start of the season when transmission is increasing (predicted mean efficacy of 64.6% compared to the true value of 52.0%), or underestimation when participants are recruited at the end of the season (49.1% versus 58.3%) (Fig 2).

### Single-strain model: Study design

**Fig 3:**
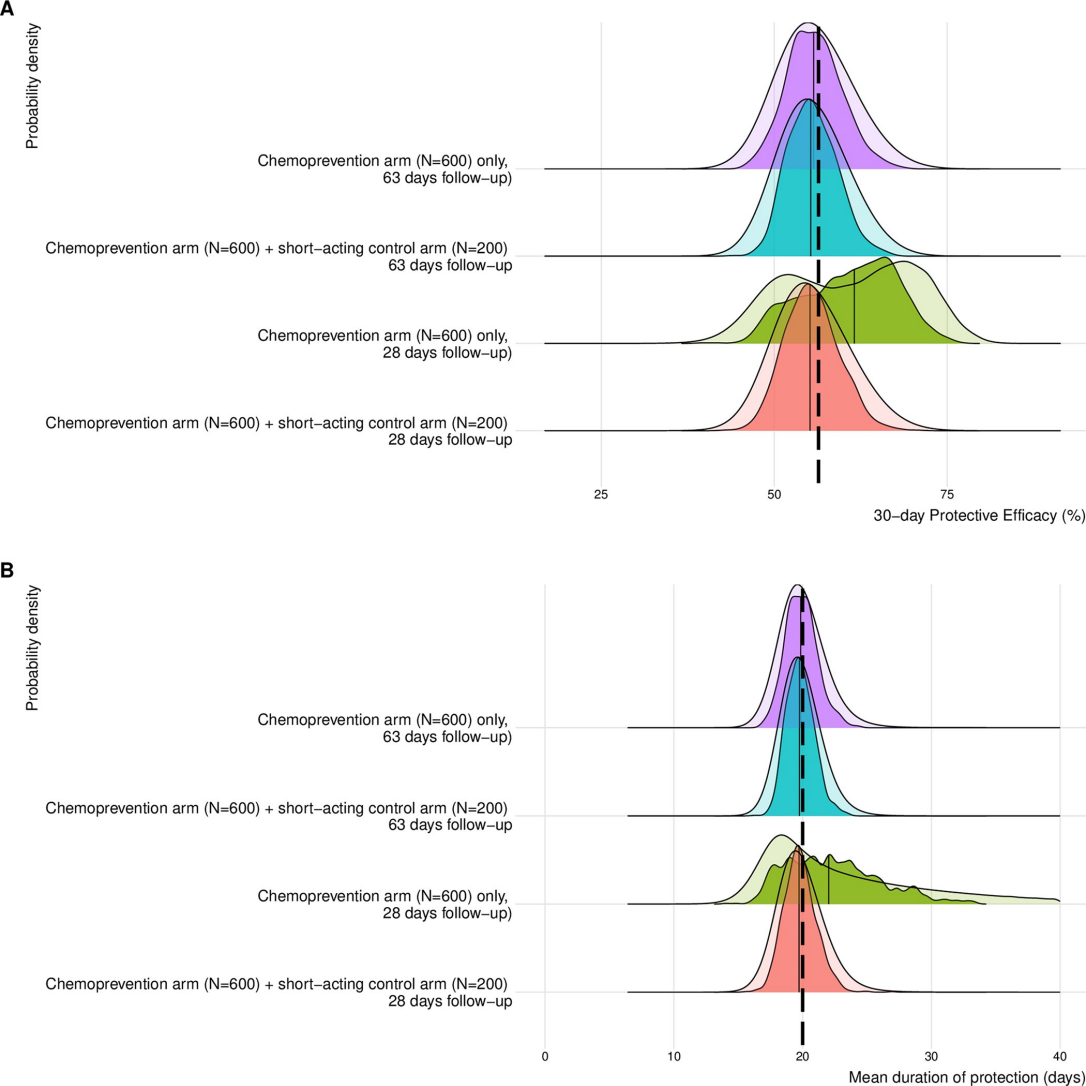
Impact of follow-up length and addition of short-acting control arm (*N* = 200) on uncertainty and precision. The probability density distribution of estimated (**A**) 30-day protective efficacy and (**B**) mean duration of protection. Protective efficacy over 30 days is calculated using the estimated posterior parameters as 1 minus the probability of being infected in the treatment arm divided by the probability of being infected in a theoretical control arm over 30 days. Mean duration of protection is also calculated using the estimated posterior parameters. The opaque probability density functions indicate the distribution of all posterior values across all 1,000 simulations (10,000 for each simulation). The darker coloured probability density functions indicate the distribution of the medians across all simulations. Vertical black lines show the median values of the estimated distributions.

We explored differences in estimated precision resulting from the addition of a control group and by control group type (Table 2 and Fig 3). The first type of control group examined is an untreated placebo group, and the second is treatment prior to day 0 with a short-acting drug that is 100% effective at clearing infections but with no post-treatment protection, such as 7 days of artesunate monotherapy [18]. Further, we explored the impact of shorter durations of follow-up on precision (42 and 28 days, compared to 63 days in the baseline scenario).

In the single-arm scenarios with follow-up of just 28 days, mean duration of protection and protective efficacy could not be precisely estimated. The median width of CrIs for 30-day protective efficacy was 27.3% (IQR = 24.3% to 29.8%) for a 28-day follow-up, compared to a much shorter width of 17.1% (14.8% to 19.9%) for a 42-day follow-up and 15.2% (IQR = 13.8% to 16.6%) for a 63-day follow-up. The respective CrI widths in mean duration of protection were 30.8 days (IQR = 25.6 to 33.5 days), 6.0 days (IQR = 4.9 to 8.2 days) and 4.8 days (IQR = 4.2 to 5.7 days). The addition of a control group with a short-acting drug (*N* = 200) substantially improves the precision in estimated efficacy and mean duration of protection estimated by a simulated trial with a 28-day follow-up (width of CrIs [IQR] was 15.5% [14.0% to 16.9%] and 5.0 days [4.4 to 5.8 days], respectively). By contrast, the addition of a control group did not make much difference to precision when the follow-up was 63 days and transmission was constant (Table 2).

A higher loss to follow-up of 20% made small differences to the precision in 30-day efficacy or duration of protection compared to 10% loss to follow-up (median width of CrI in the 20% loss to follow-up scenario was 16.0% and 5.1 days, compared to 15.2% and 4.8 days). The outputs for all scenarios are presented in S3 File and S4 File.

### Two-strain model: Transmission and study design

In a single-arm simulated trial of 600 participants, the power to detect genotype differences was only 1.4% with 28-day follow-up. The respective power for a 42-day follow-up was 71.5% and 93.5% for a 63-day follow-up (Table 3). Reducing the sample size in the baseline single-arm scenario with 63 days follow-up, to 400 individuals resulted in a power of 80.1% (Table 3). Adding a placebo control of 200 individuals (400 chemoprevention; 200 control) increased the power to 89.9%, and using a short-acting drug group in place of placebo further increased the power to 93.2%. Considering the scenario of short-acting control arm (200 participants) alongside the chemoprevention arm (400 participants) with only 42 days follow-up, increased the power from 71.5% in the single-arm trial (N_treatment_ = 600) to 90.4% in the controlled-trial (N_treatment_ = 400, N_control_ = 200). Adding a short-acting drug control group to a trial with a 28-day follow-up resulted in higher power (N_treatment_ = 400, N_control_ = 200: power = 86.6%; N_treatment_ = 600, N_control_ = 200, power = 95.5%). For relatively short/moderate follow-ups, the addition of a control group substantially improved power compared to a single-arm trial with the same total sample size. An increase in transmission intensity also improved the power to detect genotype differences (Table 3).

### Two-strain model: Assumptions on resistance

The probability that an individual is infected with a resistant parasite after chemoprevention depends not only on the probability that the drug clears the newly acquired resistant parasite, but also the genetic frequency of resistance in the parasite population (Fig 1C and 1D).

**Fig 4:**
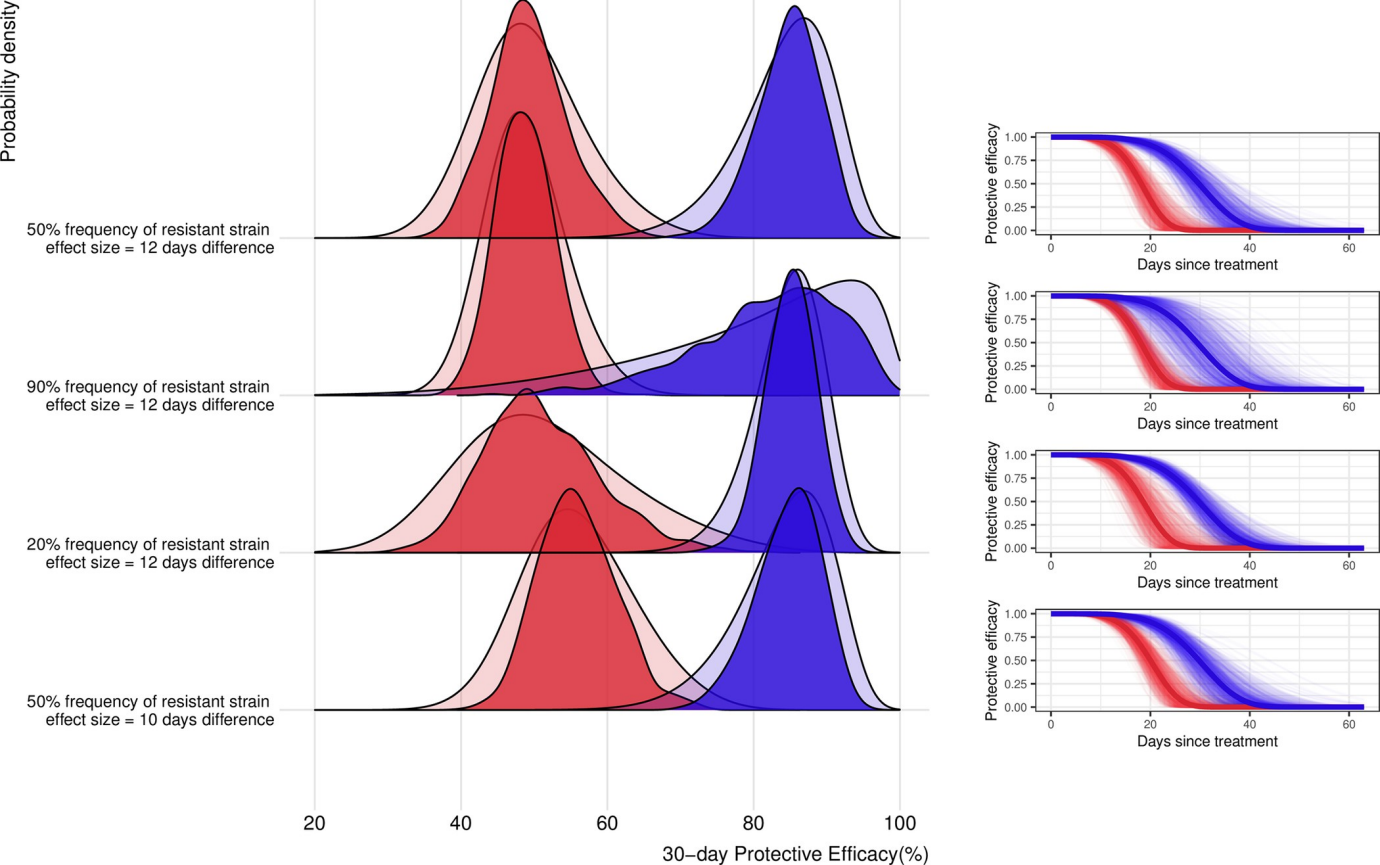
Impact of resistance genotype frequency and effect size assumptions on precision. The probability density distribution of 30-day protective efficacy against resistant (red) and sensitive (blue) strains for 4 scenarios related to the frequency of resistance and effect size are shown in the left panel. The opaque probability density functions indicate the distribution of all posterior values across all 1,000 simulations (10,000 parameter values × 1,000 simulated datasets). The darker coloured probability density functions indicate the distribution of the medians across all simulations. The panels on the right show the probability of being protected by the drug (protective efficacy) over time against the 2 strains from time since treatment. Solid lines denote the median of the medians estimated across 1,000 simulations, and faint lines show all medians estimated from a 1,000 simulations.

Power estimations are sensitive to assumptions about the underlying frequency of resistance, defined as the presence of a mutation/set of mutations of interest. When the ratio of resistant to sensitive strains is 1:1 (50% frequency of each strain), power to detect a difference in the duration of protection against the 2 strains was 93.5% in the baseline scenario (Table 3 and Fig 4). In areas where resistance is relatively rare (for instance, 20% frequency of resistant strain and 80% frequency of sensitive strain), the smaller number of resistant infections is expected to lower the estimated power (from 93.5% to 69.4%). The same is true when resistance frequency is high. For instance, when frequency of resistance is 90% power is reduced to 53.4%.

In the baseline scenario, the expected effect size was assumed to be 12 days difference in protection (30 days against sensitive parasites versus 18 days against resistant parasites). When lowering the effect size by just 2 days, the power decreased substantially from 93.5% to 83.0% (Table 3 and Fig 4). Small variations in loss to follow-up and probability of determining the strain in a diagnosed *P*. *falciparum* case have a moderate effect on power estimation (Table 3 and S4 File). Alternative power calculation methods based on Cox proportional hazards showed similar estimates for efficacy, but these methods cannot be used for testing differences in duration of drug protection or genotype differences in protective efficacy (S5 File).

Model extensions to include ≥3 strains are presented in S6 File, along with an exploration of the impact of mixed infections in the 2-strain model (S7 File). We found that the cumulative number of incident mixed infections by the last day of follow-up ranged from 0.1% to 2.4% in most simulated scenarios. The only exception was in a scenario with infrequent follow-up, 50% frequency of resistance and high incidence (10 ippy), where the cumulative percentage of mixed infections was 5.9%.

## Discussion

Chemoprevention has saved an estimated three-quarters of a million lives over the last decade [19], and its impact will grow following the WHO recommendation to scale-up programmes to a larger number of endemic countries. Accurately monitoring chemoprevention efficacy in trials, particularly in settings with drug resistance, is essential to maintaining this impact, but there are challenges in designing these trials. The novel analysis framework presented in this study identifies strengths and weaknesses in current chemoprevention trial designs and proposes methods to improve future protocols and analysis.

Our findings highlight the importance of determining the underlying incidence in the absence of drug protection during chemoprevention trials to avoid mistaking naturally occurring fluctuations in transmission levels for drug effect. In all scenarios, we confirm that protective efficacy can be estimated with greater precision in settings with higher transmission. An untreated control group is the most robust study design to measure background transmission, but withholding chemoprevention poses ethical issues. Alternatively, in areas of stable, constant transmission, we show that a long follow-up of ≥42 days allows measurement of underlying incidence when the drug is no longer active in preventing infection. For this reason, the current recommendation of 28-day follow-up in a single-arm trial may be insufficient to accurately determine protective efficacy against new infections, providing low precision. Nonetheless, the benefits of extending trial follow-up should be balanced against associated costs and feasibility, and long follow-up designs may prove demanding for participating children and their caregivers. However, once protective efficacy for different genotype profiles has been quantified, trials would not need to be repeated other than for validation, as genotype-specific protection parameters can be fixed and applied to different settings when modelling intervention impact. Even with longer follow-up, trial results could still prove inaccurate as infection risk can vary greatly over small distances or time-frames. In seasonal areas, depending on whether follow-up occurs during the start or the end of a transmission season, we show that protective efficacy and mean duration of protection may be over- or underestimated, respectively. In settings with unstable transmission, the current model can be extended to include time-varying risk of infection in analyses of controlled chemoprevention trials [12,20].

Given the ethical challenges of including a control group in a trial, WHO recommends an alternative option of a 2-arm trial including a chemoprevention group and an artesunate-lumefantrine comparator group. However, lumefantrine is expected to provide approximately 13 days protection against a new infection [7], which is likely to reduce power to detect protective efficacy in the chemoprevention group. Another option could be to use a comparator drug that is efficacious at clearing parasitaemia but is short-lived, such as 7 days of artesunate monotherapy administered before the start of follow-up [18]. Alternatively, chemoprevention could be trialled in age groups not covered by local chemoprevention strategies (i.e., >5 years for SMC or >2 years for PMC), which is likely to give a reasonable indication of efficacy in the target age group, although differences in pharmacokinetics or immunity in these groups may affect results.

Drug resistance poses one of the greatest challenges to chemoprevention, yet there are no guidelines on quantifying the impact of drug resistance on chemoprevention. Our analysis provides a method to estimate duration of protection against specific parasite genotypes, disaggregating the effects of transmission intensity, and frequency of resistant strains. This enables prediction of chemoprevention efficacy in settings with different frequencies of resistance. We find that placing a trial where both resistant and sensitive infections are common allows more precise estimation of genotype-specific protective efficacy and molecular surveillance in the trial area can provide robust estimates of the frequency of different strains [21].

A limitation of this analysis is that additional potential confounders affecting chemoprevention efficacy, such as drug absorption, metabolism, drug quality, nonadherence, coverage/access, acquired immunity, and level of parasitaemia were not considered [10,22–26]. For instance, a placebo-controlled chemoprevention trial of SP-AQ showed higher efficacy in children aged under 2 compared to children aged 2 to 5 [27], though this age effect was not significant in other studies [28]. Another challenge of this work is that sample size estimations for estimating duration of drug protection require high-performance computing, particularly when a broad range of sample sizes/scenarios need to be assessed.

This analysis focuses on incidence of malaria infection rather than clinical malaria incidence, which is an important outcome in quantifying malaria burden and assessing the value of chemoprevention. The probability of becoming symptomatic depends on the levels of immunity acquired after repeated exposure to malaria and therefore varies with respect to the age-range of participants, transmission level, and malaria intervention coverage [29,30]. If only 50% of new malaria infections become symptomatic, the power to detect genotype differences in the duration of protection from a clinical infection would reduce to 79.5%, compared to 93.5% for any malaria infection, assuming that the probability of an infection becoming symptomatic is unaffected by chemoprevention [31].

In the approach modelled, the power is reduced by excluding those who are positive on day 0. Retaining these individuals will preserve statistical power but may introduce bias due to inability to differentiate between existing and new infections. Standard PCR amplification of mixtures of alleles lacks the sensitivity for assessing treated asymptomatic infections and cannot reliably detect alleles present at low frequencies, leading to potential misclassification of recurrent infections [32,33]. This is particularly important for SP, where recrudescence may be common in many areas of high SP-resistance.

The approach presented here can inform the design and analysis of chemoprevention trials to drive national policy decision-making, such as informing selection of sites with particular characteristics that optimise power for a given sample size in the presence of budget constraints or other logistical challenges. This modelling approach has been applied in the design of parasite clearance and protection against infection (PCPI) studies aiming to look at genotype specific effects on chemoprevention efficacy of SP [13]. Additionally, our method can produce essential inputs for decision-making relevant to malaria chemoprevention. Quantifying protective efficacy provided by different implementations can help inform policy decisions that are tailored to specific countries or subnational areas. For instance, estimates of protective efficacy by genotype (in combination with up-to-date area-surveillance of mutation prevalence) can be incorporated into tools that national malaria control programmes can use to predict the likely impact of these interventions.

## Supporting information

S1 File Supplementary methods. (DOCX)

S2 File Relationship between prevalence and incidence of infection and clinical malaria. (DOCX)

S3 File One-strain model. (DOCX)

S4 File Two-strain model. (DOCX)

S5 File Three-strain model. (DOCX)

S6 File Extension of model to include incident mixed infections. (DOCX)

S7 File Power calculation using the Cox proportional hazards method. (DOCX)

S8 File Summary table of key messages. (DOCX)

## Abbreviations

ACT: artemisinin-combination therapy
AQ: amodiaquine
CPES: chemoprevention efficacy study
CrI: credible interval
ippy: infections per person per year
IPTp: intermittent preventative treatment in pregnancy
IPTsc: intermittent preventive treatment in school-aged children
MCMC: Markov Chain Monte Carlo
MDA: mass drug administration
PCPI: parasite clearance and protection against infection
PDMC: post-discharge malaria chemoprevention
PMC: perennial malaria chemoprevention
rfMDA: reactive focal MDA
SMC: seasonal malaria chemoprevention
SP: sulfadoxine-pyrimethamine
WHO: World Health Organisation
