# Supplementary material for: Measuring protective efficacy and quantifying the impact of drug resistance: A novel malaria chemoprevention trial design and methodology
Source: PLoS Med. 2024 May 9;21(5):e1004376. doi: 10.1371/journal.pmed.1004376 (PMC11081503; doi:10.1371/journal.pmed.1004376)
Supplement: S1 File — (DOCX) [file pmed.1004376.s001.docx]

# S1 File - Supplementary Methods

## 1.Deterministic Model

### 1.1.One-strain

In a discrete-time model we coded the probability of infection between time *t* and $\left( t-dt \right)$, where $dt$ is the time interval. In the absence of chemoprevention, the probability of a new infection, $P_{inf}$, is assumed to be constant in perennial settings and is determined by:

$$P_{inf}=1-e^{\left( -\Lambda dt \right)},$$

where Λ is the incidence of infection (instantaneous rate). The infection rate ($\Lambda$), is dependent on the EIR and the probability that an infectious bite leads to a successful infection.

By discretising over time, the cumulative proportion of individuals who are infected $I$ or remain uninfected $U$ at each time point $\left( t \right)$, can be described as:

$I\left( t \right)=I\left( t-dt \right)+I_{new}\left( t \right)$,

$$U\left( t \right)=U\left( t-dt \right)-I_{new}\left( t \right)$$

In the *presence* of chemoprevention, the probability that an individual gets infected during time step *t* since receiving chemoprevention (*t*) is modelled as:

$$P_{{inf}_{C}}\left( t \right)=P_{inf}\left( 1-\delta(t) \right)$$

Where δ is the probability of protection by the drug.

$$\delta=e^{{-\left( \frac{t}{\lambda} \right)}^{w}}$$

and the proportion with a new infection on day t following chemoprevention can be expressed as:

$$I_{new}\left( t \right)=U\left( t-dt \right) P_{{inf}_{C}}\left( t \right)$$

### 1.2.Two-strain

In the *absence* of chemoprevention, the probability that an individual becomes infected with each strain depends on the frequency of resistant strain ($F_{R}$):

$$P_{{inf}_{R}}=P_{inf}F_{R} ,$$

$$P_{{inf}_{S}}=P_{inf}(1-F_{R}) ,$$

The probability that an individual becomes infected with each strain at each time step (between t and t-1) following chemoprevention was estimated as:

$$P_{{inf}_{R_{C}}}\left( t \right)=P_{{inf}_{R}}\left( 1-\delta_{R} \right) ,$$

$$P_{{inf}_{S_{C}}}\left( t \right)=P_{{inf}_{S}}\left( 1-\delta_{S} \right) ,$$

Where,
$\delta_{R}=e^{{-\left( \frac{t}{\lambda_{R}} \right)}^{w_{R}}}$ = probability of drug protection against the resistant strain, and

$\delta_{S}=e^{{-\left( \frac{t}{\lambda_{S}} \right)}^{w_{S}}}$ = probability of drug protection against the sensitive strain.

The equations describing the cumulative proportion of individuals who are infected with the resistant strain ($I_{R})$, infected with the sensitive strain${(I}_{S})$, or uninfected ($U$), over time are:

$I_{R}\left( t \right)=I_{R}\left( t-dt \right)+I_{R new}\left( t \right)$,

$I_{S}\left( t \right)= I_{S}\left( t-dt \right)+I_{S new}\left( t \right)$,

$U\left( t \right)=U\left( t-dt \right)-I_{R new}\left( t \right)-I_{S new}\left( t \right)$,

where $I_{R new}\left( t \right)$ and $I_{S new}\left( t \right)$ is the proportion of newly acquired proportions at each time point and can be expressed as:

$I_{R new}\left( t \right)=U\left( t-dt \right)P_{{inf}_{R_{C}}}\left( t \right)$,

$$I_{S new}\left( t \right)= U\left( t-dt \right)P_{{inf}_{S_{C}}}\left( t \right)$$

The number of those who remain uninfected and new infections with each parasite strain at each time point are assumed to be multinomially distributed with probabilities $\frac{U\left( t \right)}{U\left( t-dt \right)}$, $\frac{I_{R}\left( t \right)-I_{R}\left( t-dt \right)}{U\left( t-dt \right)}$ , and $\frac{I_{S}\left( t \right)-I_{S}\left( t-dt \right)}{U\left( t-dt \right)}$.

The diagram below shows the model structure and how chemoprevention affects the natural history of disease.


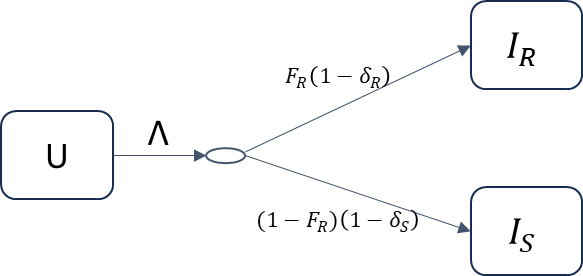


The two-strain model was extended to account for the fact that some infections will not be genotyped at a particular codon position due to PCR failure and loss of genetic material. We assume that this probability is not associated with the presence of resistance. The probability of being determined for a specific genotype (or haplotype), denoted by $p_{determ}$ can be incorporated as follows:

$$I_{R\_determ\_new}\left( t \right)=U\left( t-dt \right)\times P_{{inf}_{R_{C}}}\left( t \right)\times p_{determ}$$

$$I_{S\_determ\_new}\left( t \right)= U\left( t-dt \right)\times P_{{inf}_{S_{C}}}\left( t \right)\times p_{determ}$$

$$I_{undeterm\_new}\left( t \right)=\left( U\left( t-dt \right)\times P_{{inf}_{R_{C}}}\left( t \right)+U\left( t-dt \right)\times P_{{inf}_{S_{C}}}\left( t \right) \right)\times(1-p_{determ})$$

$$I_{R\_determ}\left( t \right)=I_{R\_determ}\left( t-dt \right)+I_{R\_determ\_new}\left( t \right)$$

$$I_{S\_determ}\left( t \right)= I_{S\_determ}\left( t-dt \right)+I_{S\_determ\_new}\left( t \right)$$

$$I_{undeterm}\left( t \right)=I_{undeterm}\left( t-dt \right)+I_{undeterm\_new}\left( t \right)$$

$$U\left( t \right)=U\left( t-dt \right)-I_{R\_determ\_new}\left( t \right)-I_{S\_determ\_new}\left( t \right)-I_{undeterm\_new}\left( t \right))$$

The number of new infections determined with each parasite strain, the number of infections with undetermined genotype and those who remain uninfected in the interval (t – 1,t] are assumed to be multinomially distributed with probabilities $\frac{I_{R\_determ}\left( t \right)-I_{R\_determ}\left( t-dt \right)}{U\left( t-dt \right)}$ , $\frac{I_{S\_determ}\left( t \right)-I_{S\_determ}\left( t-dt \right)}{U\left( t-dt \right)}$ , $\frac{I_{undeterm}\left( t \right)-I_{undeterm}\left( t-dt \right)}{U\left( t-dt \right)}$ and $\frac{U\left( t \right)}{U\left( t-dt \right)}$. The diagram below shows this extension.


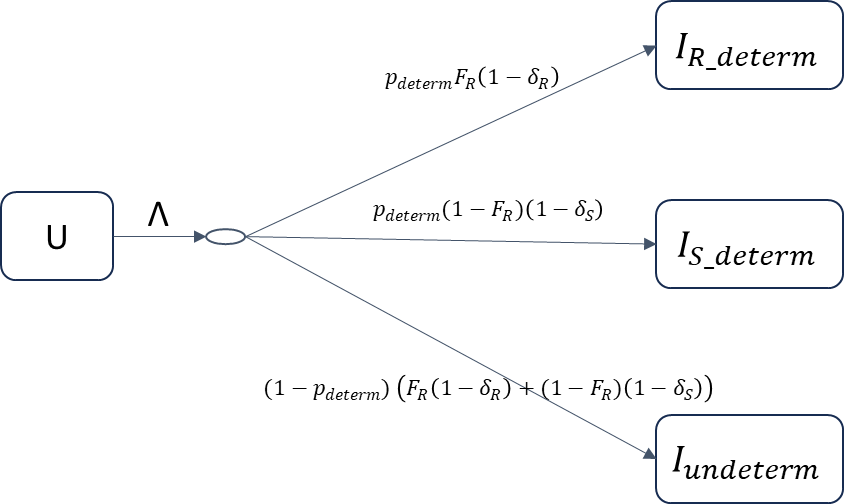


The 30-day efficacy (overall and by strain) was estimated as the percentage of new infections (with strain X) prevented by the drug compared to no chemoprevention:

$1-\frac{I_{day30 (treated)}}{I_{day30 (untreated)}}$,

Where $I_{day30}$ is the cumulative proportion infected by day 30 in each arm.

## 2.Simulations: inputs and assumptions

To investigate the effect of different epidemiological settings and study designs on estimation of chemoprevention efficacy, we generated stochastic simulations of a large number of trials with one or two strains. At the start of the simulation, all individuals are uninfected (state U) and are at risk of a new infection.

The stochastic processes include the probability of being exposed to the parasite, that exposure is either to a resistant/sensitive parasite, and that exposure to each genotype results in a successful infection. A baseline scenario of 600 children was considered, where children who are negative for *P. falciparum* on day 0 are analysed for protection from a new infection. The reason for excluding day 0 positives is due to difficulty in accurately determining which genotypes are due to a *new* infection or an *existing* infection. The baseline scenario assumes a slide prevalence of 40%, an infection rate of 10 ippy and a loss-to-follow-up of 10%(1). In the simulation we assume that those lost to follow-up are not analysable and are removed from those at risk on day 0, providing a more conservative estimate of power. In the single-strain model, we assume the drug provides a 20-day protection against any parasite and, for the two-strain model, a 30-day duration of protection against sensitive parasites (without the mutation of interest) and an 18-day duration of protection against resistant parasites (effect size of 12 days). Further, the frequency of the resistant strain in the parasite population was assumed to be 50%, and that 90% of confirmed infections will be successfully genotyped at the position of interest. In the baseline scenario, participants are followed up over 9 weeks at multiple time points (days 0,2,3,5,7,14,21,28,35,42,49, 56, and 63).

In the simulations, different scenarios were explored by varying input parameters. These included variations in the incidence/prevalence of infection, seasonality, loss to follow-up, length of follow-up, sample size and the presence of a control group. For comparability purposes, in scenarios reflecting seasonality in transmission, we assumed a change in incident infections over follow-up, with mean incidence of malaria being the same as that in the baseline scenario (constant risk), and the minimum and maximum incidence being 50% lower/higher compared to that constant. Additionally, for the one-strain model, we explored different durations of protection against any parasite; for the two-strain model, we varied the frequency of resistance, the expected duration of protection against the resistant strain, and the probability of successfully genotyping an infection. For each of these scenarios, we generated 1000 simulated datasets. The analysis showed that this number of simulations was sufficient as the deterministic model proportions closely matched the median values across all simulations. Simulated datasets contained the number of individuals who remained uninfected, and those who were newly infected (with any or a sensitive/resistant parasite) at each time step.

**References**

1. Zani B, Gathu M, Donegan S, Olliaro PL, Sinclair D. Dihydroartemisinin-piperaquine for treating uncomplicated *Plasmodium falciparum* malaria. Cochrane Database Syst Rev. 2014;2014(1):Cd010927.
