# Supplementary material for: Measuring protective efficacy and quantifying the impact of drug resistance: A novel malaria chemoprevention trial design and methodology
Source: PLoS Med. 2024 May 9;21(5):e1004376. doi: 10.1371/journal.pmed.1004376 (PMC11081503; doi:10.1371/journal.pmed.1004376)
Supplement: S2 File — (DOCX) [file pmed.1004376.s002.docx]

# S2 File - Relationship between prevalence and incidence of infection and clinical malaria.


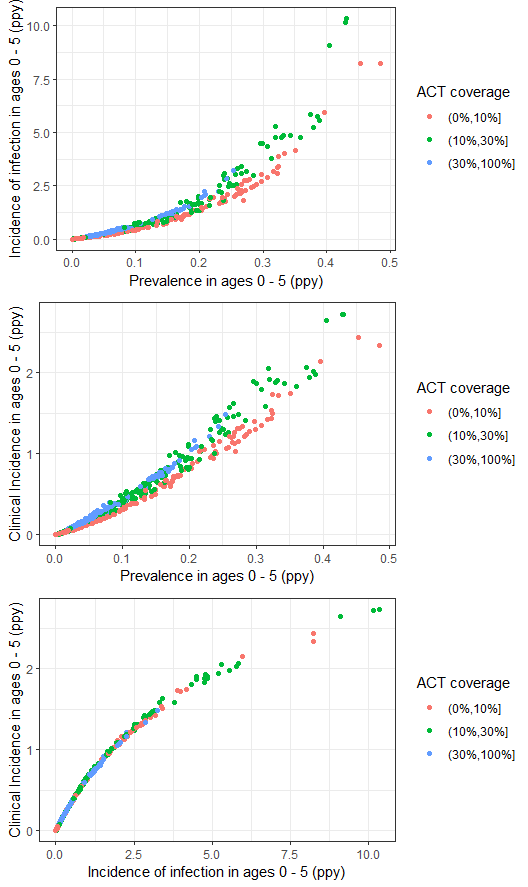


iii)

ii)

i)

Fig A - The relationship between parasite prevalence by microscopy and (i) incidence of infection in 0-5 year olds, and (ii) clinical incidence in 0-5 year olds. Panel iii shows the relationship between incidence of infection and clinical incidence in 0-5 year olds. These panels show predictions from a malaria transmission model developed by Imperial College. Each dot represents a first-level administrative unit in sub-Saharan Africa. Details of the model structure and fitting can be found in Griffin 2010 (<https://doi.org:10.1371/journal.pmed.1000324>), and Griffin 2014 (https://doi.org:10.1038/ncomms4136)
