# Supplementary material for: Measuring protective efficacy and quantifying the impact of drug resistance: A novel malaria chemoprevention trial design and methodology
Source: PLoS Med. 2024 May 9;21(5):e1004376. doi: 10.1371/journal.pmed.1004376 (PMC11081503; doi:10.1371/journal.pmed.1004376)
Supplement: S3 File — (DOCX) [file pmed.1004376.s003.docx]

# S3 File - One-strain model

Table A - Scenarios and parameter inputs for the single-strain model in addition to those shown in Table 1. The 30-day protective efficacy is the deterministic value based on the input parameters shown in the table.

| **Varying parameter** | **Sample size**  **(N)** | **Length of follow-up (days)** | **Mean Incidence (ippy*)** | **Seasonality** | **Slide Prevalence (%)** | **Loss to follow-up (%)** | **Mean duration of protection (days)** | **30-day protective efficacy (%)** |
| --- | --- | --- | --- | --- | --- | --- | --- | --- |
| **Baseline scenario** | **600** | **63** | **10** | **none** | **40** | **10** | **20** | **56.4** |
| ↓protection | 600 | 63 | 10 | none | 40 | 10 | **15** | 39.1 |
| ↑protection | 600 | 63 | 10 | none | 40 | 10 | **25** | 74.1 |
| Addition of untreated control group** | **600+200** | 63 | 10 | none | 40 | 10 | **20** | 56.4 |
| Addition of untreated control group  ↓Sample size for chemoprevention | **400+200** | 63 | 10 | none | 40 | 10 | 20 | 56.4 |
| Addition of short-acting clearance drug group  ↓Sample size for chemoprevention | **400+200** | 63 | 10 | none | 40 | 10 | 20 | 56.4 |
| Addition of short-acting clearance drug group  ↓Sample size for chemoprevention  ↓Length of follow-up | **400+200** | **42** | 10 | none | 40 | 10 | 20 | 56.4 |
| Addition of short-acting clearance drug group  + seasonality(start) | **600+200** | 63 | 10 | **start** | 40 | 10 | 20 | 52.0 |
| Addition of short-acting clearance drug group  + seasonality(end) | **600+200** | 63 | 10 | **end** | 40 | 10 | 20 | 58.3 |
| ↑loss to follow-up | 600 | 63 | 10 | none | 40 | **20** | 20 | 56.4 |
| *ippy = infections per person per year  **If participants are excluded based on being parasite -positive on day 0, this reduces the sample size for analysis, resulting in lower precision for the same incidence. However, parasite prevalence on day 0 and infection incidence are highly correlated, with a likely trade-off between the two in terms of precision | | | | | | | | |


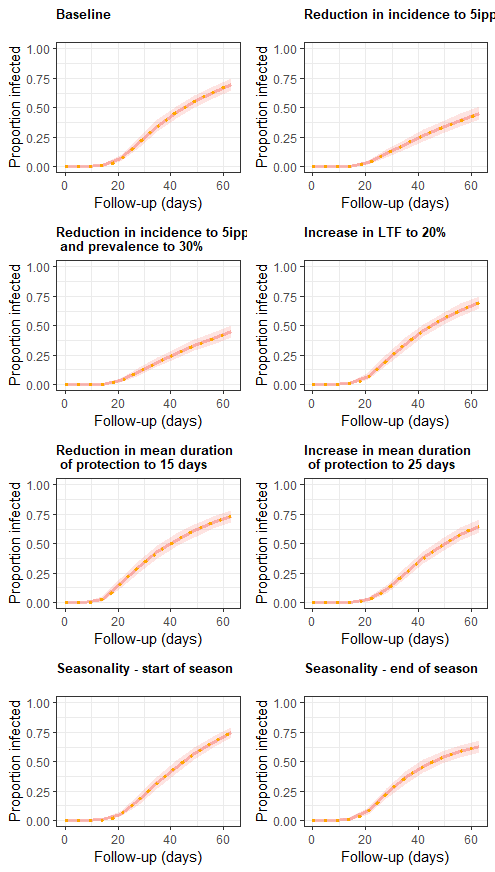


Fig A – The distribution of the proportion of new infections over time since treatment in the simulated data, across 1000 simulations for each scenario related to setting characteristics and effect size. The solid lines denote the median, and shaded areas show the 2.5th and 97.5th percentiles. The overlaid dotted line shows the expected outputs from a deterministic model with no stochasticity, which aligns with the medians across 1000 simulations.


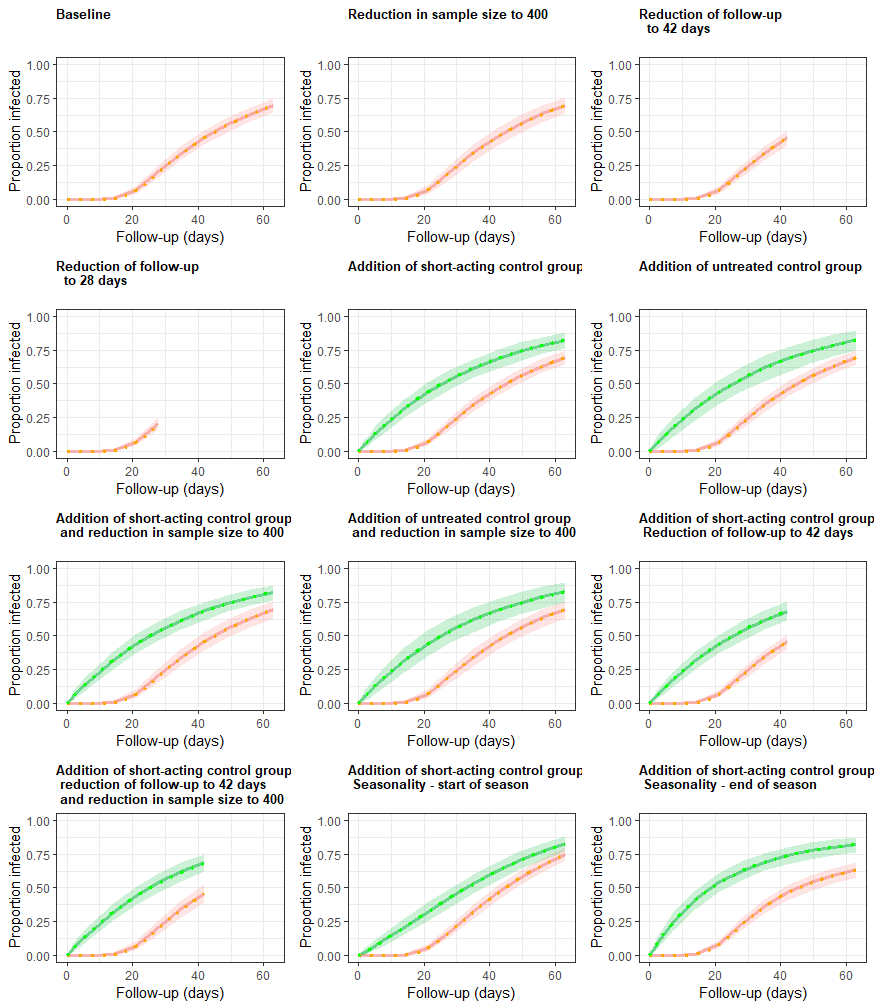


Fig B - The distribution of the proportion of new infections over time since treatment in the simulated data, across 1000 simulations for each scenario related to study design (sample size, follow-up, and presence of control group (in green)). The solid lines denote the median, and shaded areas show the 2.5th and 97.5th percentiles. The overlaid dotted line shows the expected outputs from a deterministic model with no stochasticity, which aligns with the medians across 1000 simulations.


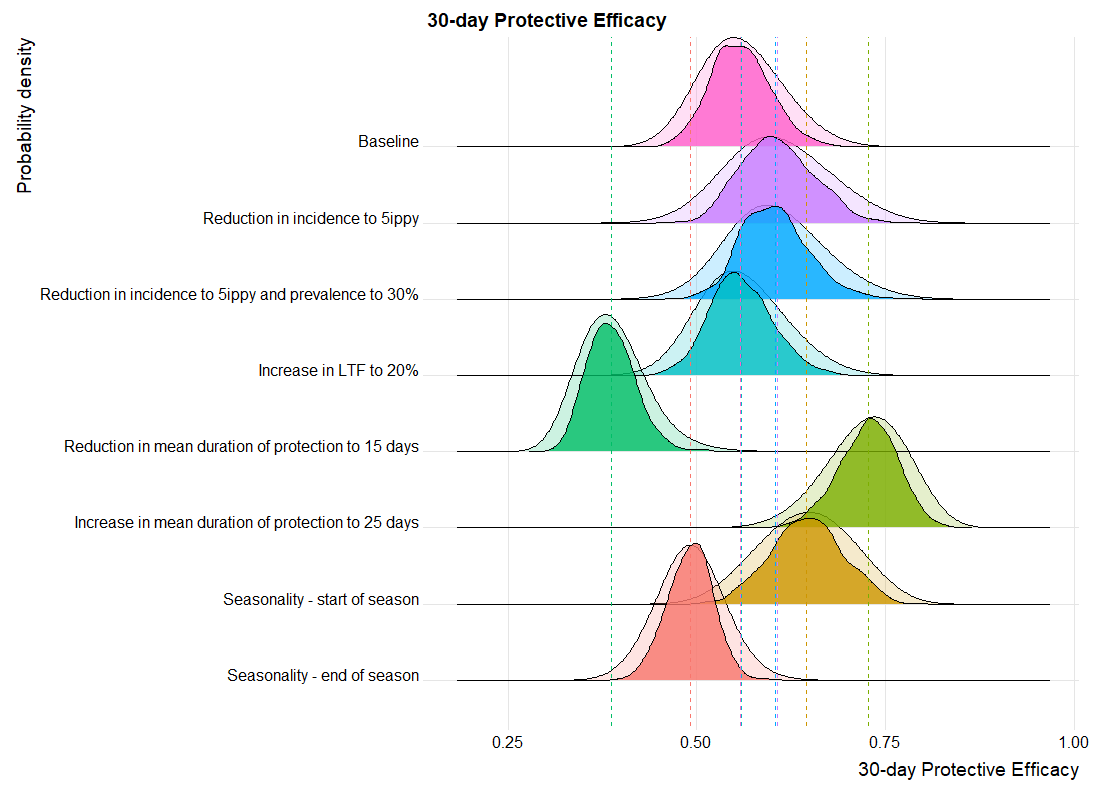


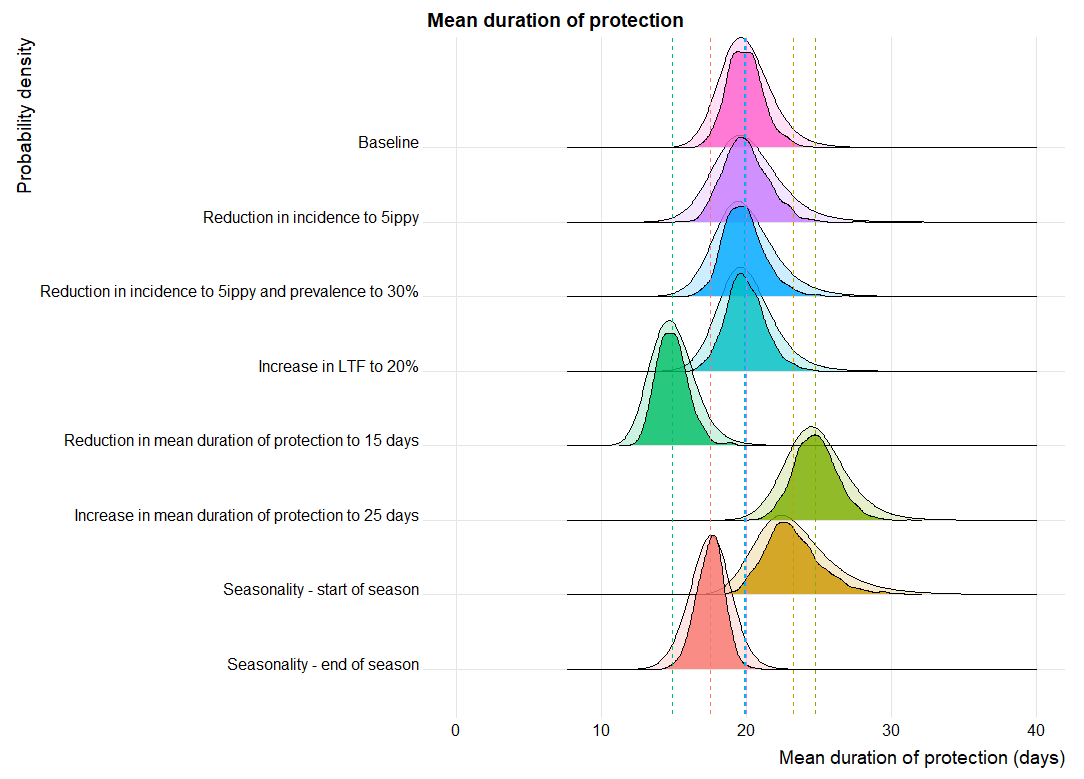


Fig C - The probability density distribution of estimated (A) 30-day protective efficacy and (B) mean duration of protection in relation to setting characteristics, loss to follow-up and expected effect size. Protective efficacy over 30 days is calculated using the estimated posterior parameters as 1 minus the probability of being infected in the treatment arm divided by the probability of being infected in a theoretical control arm over 30 days. Mean duration of protection is also calculated using the estimated posterior parameters. The opaque histograms indicate the distribution of all posterior values across all 1000 simulations (10,000 for each simulation). The darker coloured histograms indicate the distribution of the medians across all simulations.


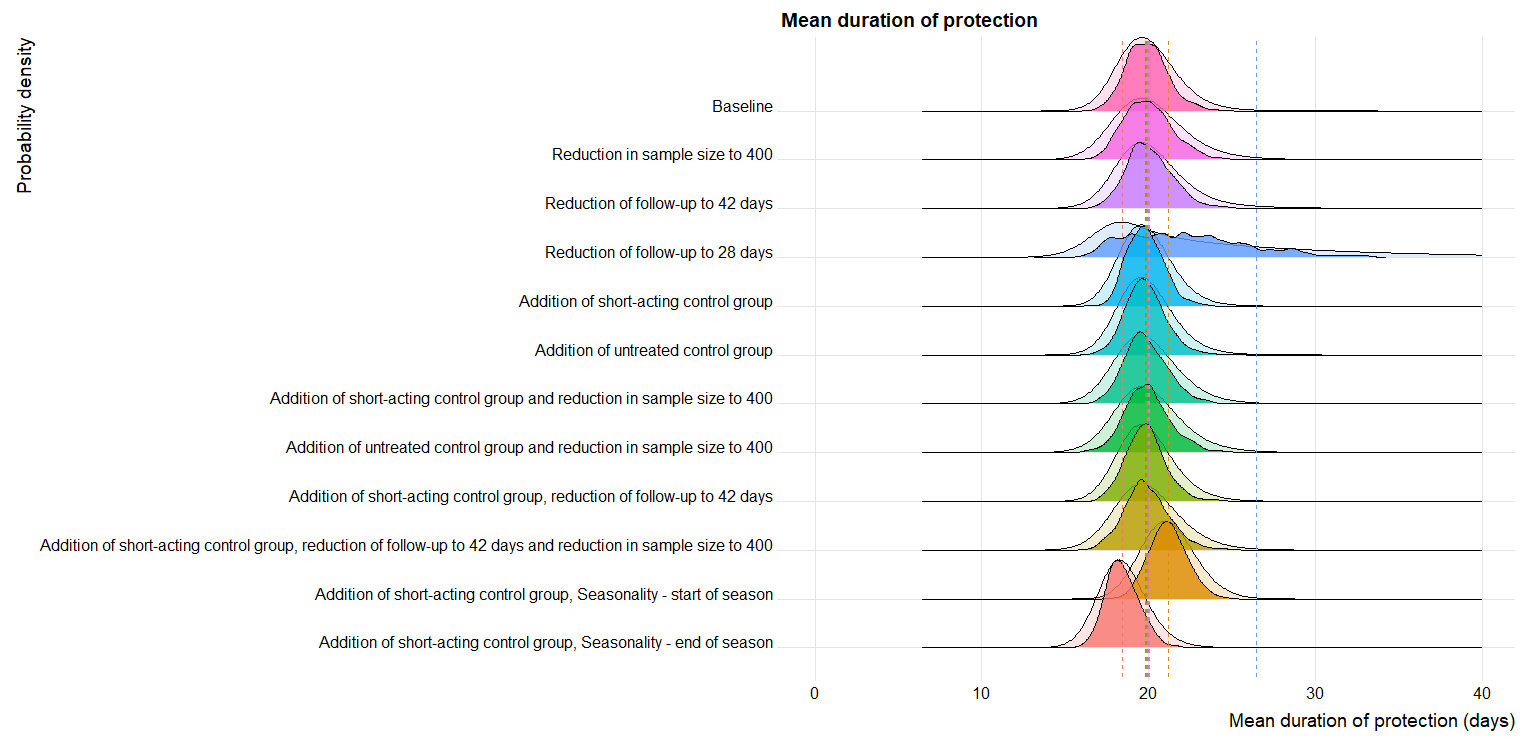


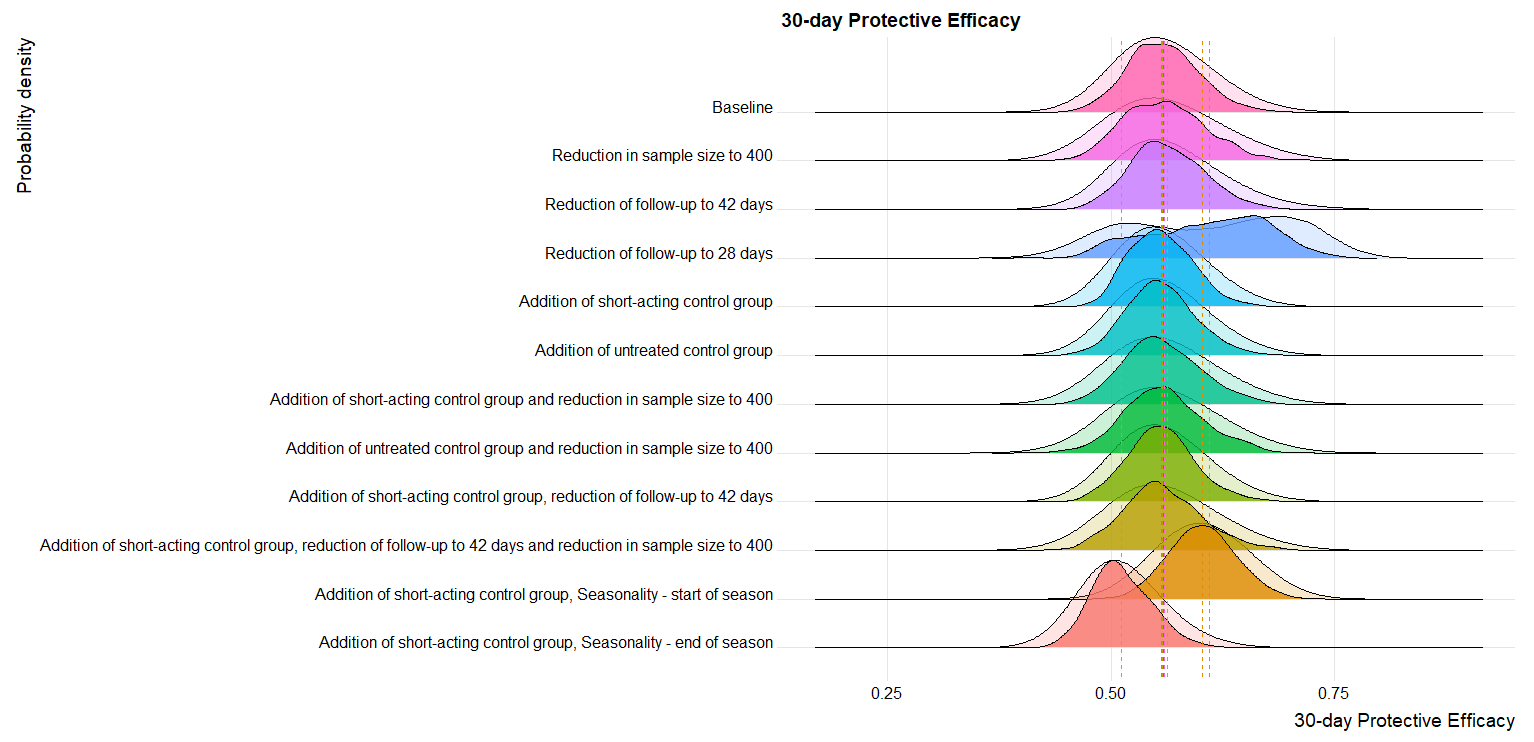
Fig D - The probability density distribution of estimated (A) 30-day protective efficacy and (B) mean duration of protection in relation to study design in perennial (baseline) and seasonal settings. Protective efficacy over 30 days is calculated using the estimated posterior parameters as 1 minus the probability of being infected in the treatment arm divided by the probability of being infected in a theoretical control arm over 30 days. Mean duration of protection is also calculated using the estimated posterior parameters. The opaque histograms indicate the distribution of all posterior values across all 1000 simulations (10,000 for each simulation). The darker coloured histograms indicate the distribution of the medians across all simulations.


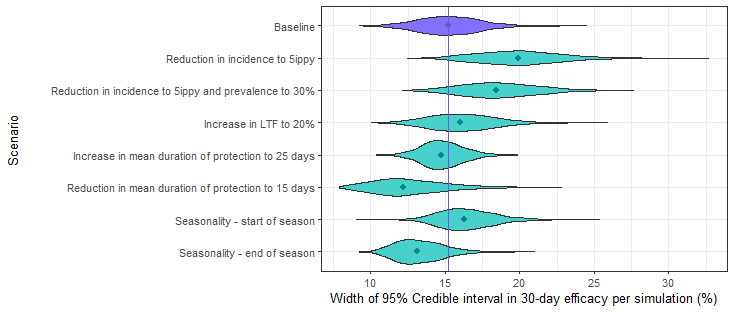


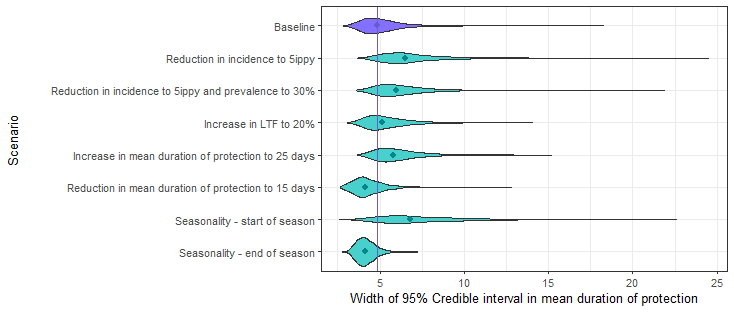


Fig E - Violin plots indicating precision in estimating 30-day protective efficacy (top) and mean duration of protection (bottom) for setting characteristics and expected effect size. The distribution of the width 95^th^ credible interval in protective efficacy shown is across 1000 simulations (simple model). Dots represent the median width across all simulations.


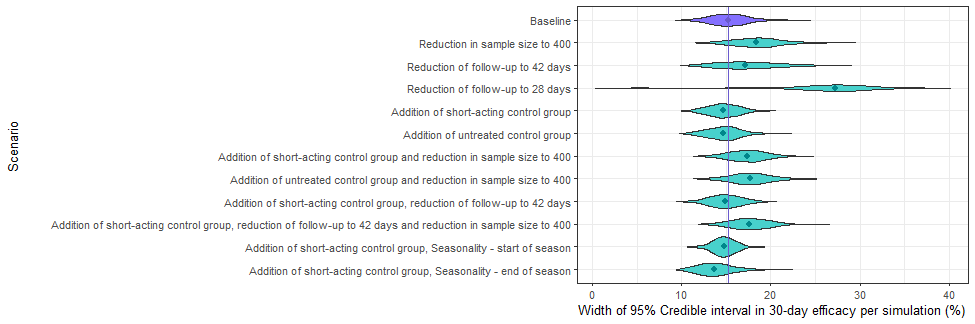


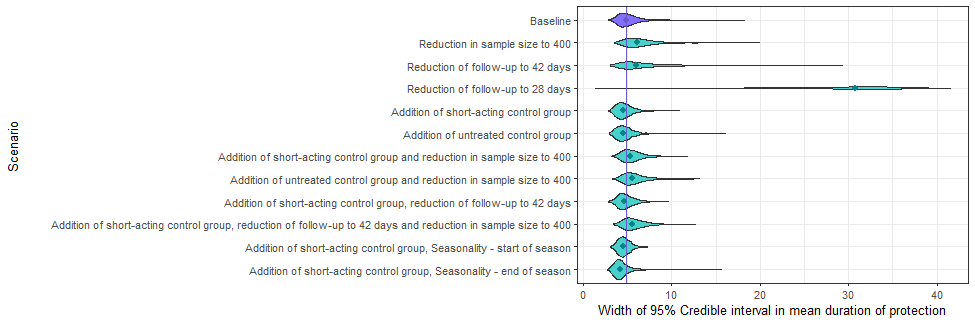


Fig F - Violin plots indicating precision in estimating 30-protective efficacy (top) and mean duration of protection (bottom) for variations in study design. The distribution of the width 95^th^ credible interval in protective efficacy shown is across 1000 simulations (simple model). Dots represent the median width across all simulations.
