# Supplementary material for: Measuring protective efficacy and quantifying the impact of drug resistance: A novel malaria chemoprevention trial design and methodology
Source: PLoS Med. 2024 May 9;21(5):e1004376. doi: 10.1371/journal.pmed.1004376 (PMC11081503; doi:10.1371/journal.pmed.1004376)
Supplement: S4 File — (DOCX) [file pmed.1004376.s004.docx]

# S4 File - Two-strain model

## Table A - Scenarios and parameter inputs for the two-strain model in addition to Table 2.

The power to detect a significant difference in the mean duration of protection (% of simulations that reject the null hypothesis of no difference) is shown in the table; R= resistant strain, S= sensitive strain. For additional scenarios, see Supplementary Table 1.

| **Varying parameter** | **Sample size**  **(N)** | **Length of follow-up**  **(days)** | **Mean Incidence (ippy*)** | **Slide Prevalence (%)** | **Freq of R (%)** | **Infections determined as R or S (%)** | **Loss to follow-up (%)** | **Mean duration of protection against S (days)** | **Mean duration of protection against R (days)** | **Effect size (days)** | **Power (%)** |
| --- | --- | --- | --- | --- | --- | --- | --- | --- | --- | --- | --- |
|  |  |  |  |  |  |  |  |  |  |  |  |
| **Baseline scenario** | **600** | **63** | **10** | **40** | **50** | **90** | **10** | **30** | **18** | **12** | 93.5 |
|  | | | | | | | | | | | |
| ↓Incidence | 600 | 63 | **5** | 40 | 50 | 90 | 10 | 30 | 18 | 12 | 73.3 |
| ↓Incidence and ↓prevalence | 600 | 63 | **5** | **30** | 50 | 90 | 10 | 30 | 18 | 12 | 78.8 |
|  |  |  |  |  |  |  |  |  |  |  |  |
| ↑loss to follow-up | 600 | 63 | 10 | 40 | 50 | 90 | **20** | 30 | 18 | 12 | 89.3 |
|  |  |  |  |  |  |  |  |  |  |  |  |
| ↓% infections with genotype determined | 600 | 63 | 10 | 40 | 50 | **70** | 10 | 30 | 18 | 12 | 89.2 |
|  |  |  |  |  |  |  |  |  |  |  |  |
| ↓Length of follow-up | 600 | **42** | 10 | 40 | 50 | 90 | 10 | 30 | 18 | 12 | 71.5 |
| ↓↓Length of follow-up | 600 | **28** | 10 | 40 | 50 | 90 | 10 | 30 | 18 | 12 | 1.4 |
| Addition of untreated control group | **600+200** | 63 | 10 | 40 | 50 | 90 | 10 | 30 | 18 | 12 | 97.1 |
| Addition of short-acting clearance drug group | **600+200** | 63 | 10 | 40 | 50 | 90 | 10 | 30 | 18 | 12 | 97.7 |
| Addition of short-acting clearance drug group  ↓Length of follow-up | **600+200** | **42** | 10 | 40 | 50 | 90 | 10 | 30 | 18 | 12 | 96.6 |
| Addition of short-acting clearance drug group  ↓↓Length of follow-up | **600+200** | **28** | 10 | 40 | 50 | 90 | 10 | 30 | 18 | 12 | 95.5 |
| *ippy = infections per person per year | | | | | | | | | | | |


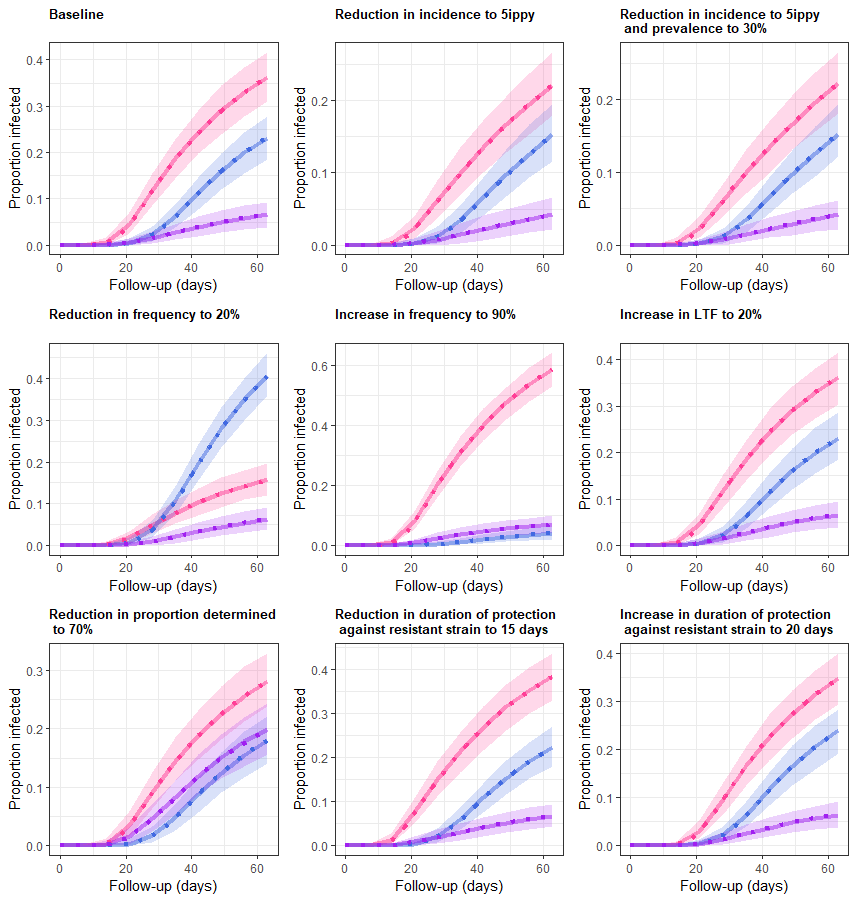


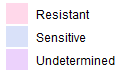


Fig A - The distribution of the proportion of new infections with undetermined genotype, and the proportion of new infections that are with a resistant and sensitive parasite in the simulated data, across 1000 simulations for each scenario related to setting characteristics and effect size. The solid lines denote the median, and shaded areas show the 2.5th and 97.5th percentiles. The overlaid dotted line shows the expected outputs from a deterministic model with no stochasticity, which aligns with the medians across 1000 simulations.


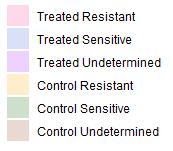

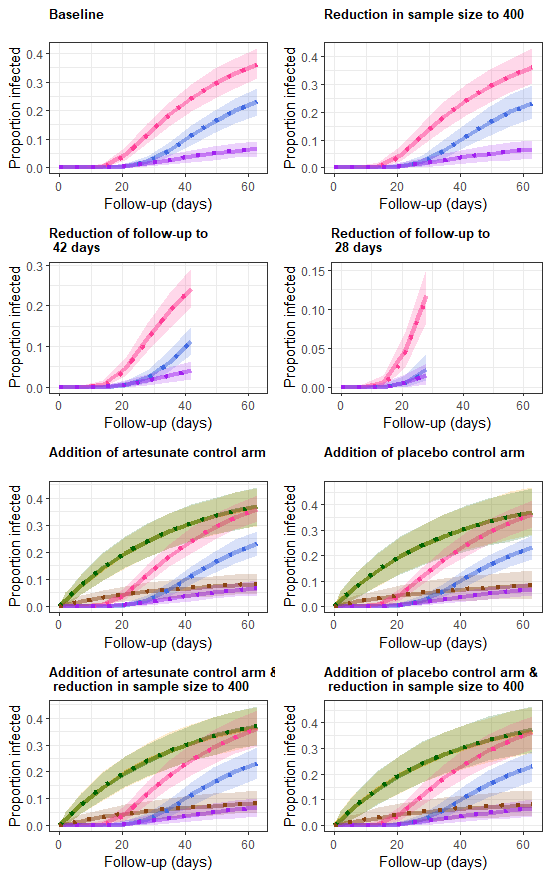


Fig B - The distribution of the proportion of new infections with undetermined genotype, and the proportion of new infections that are with a resistant and sensitive parasite in the simulated data, across 1000 simulations for each scenario related to study design. The solid lines denote the median, and shaded areas show the 2.5th and 97.5th percentiles. The overlaid dotted line shows the expected outputs from a deterministic model with no stochasticity, which aligns with the medians across 1000 simulations. Note that the yellow “control resistant” overlaps with green “control sensitive”, as frequency of resistance is 50%)


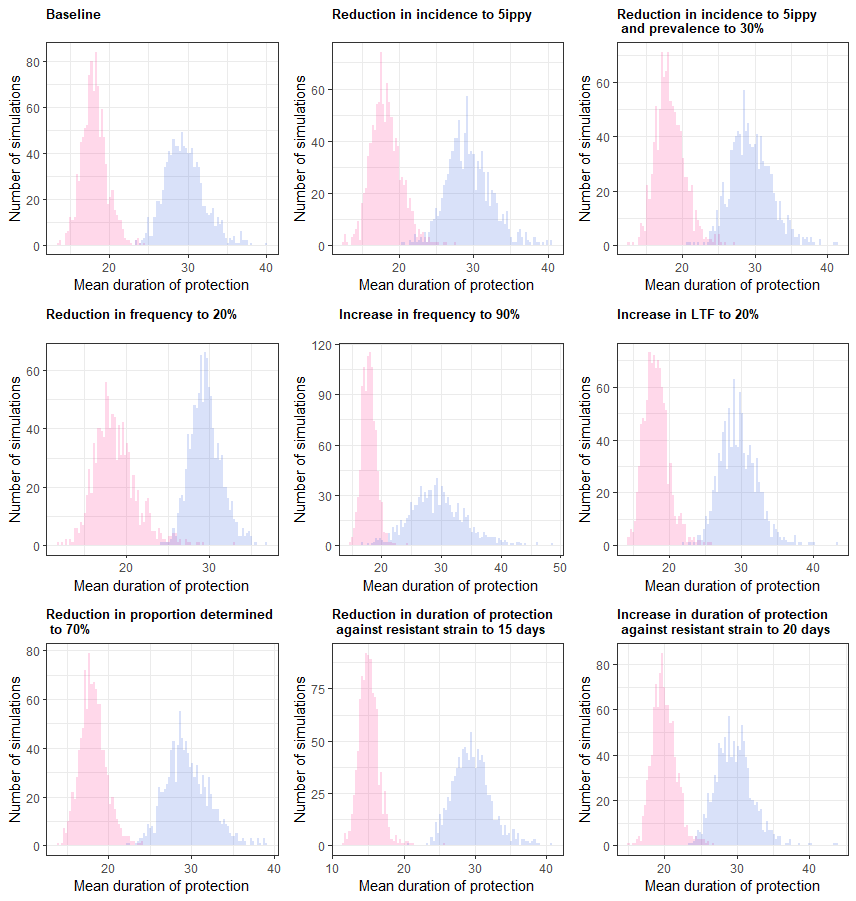


Fig C – Histograms showing the median posterior value for the mean duration of protection for resistant (pink) and sensitive (blue) strains for each scenario related to setting characteristics and effect size. Note that this shows variability *between* simulations in median posterior estimates rather than *within* a single simulation.


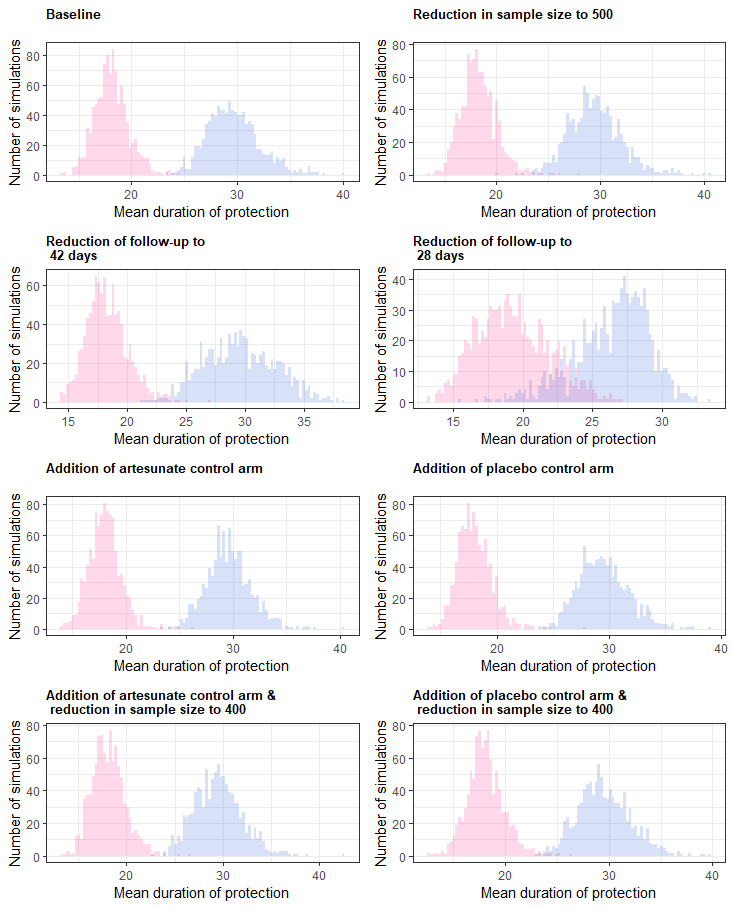


Fig D – Histograms showing the median posterior value for the mean duration of protection for resistant (pink) and sensitive (blue) strains for each scenario related to study design. Note that this shows variability *between* simulations in median posterior estimates rather than *within* a single simulation.


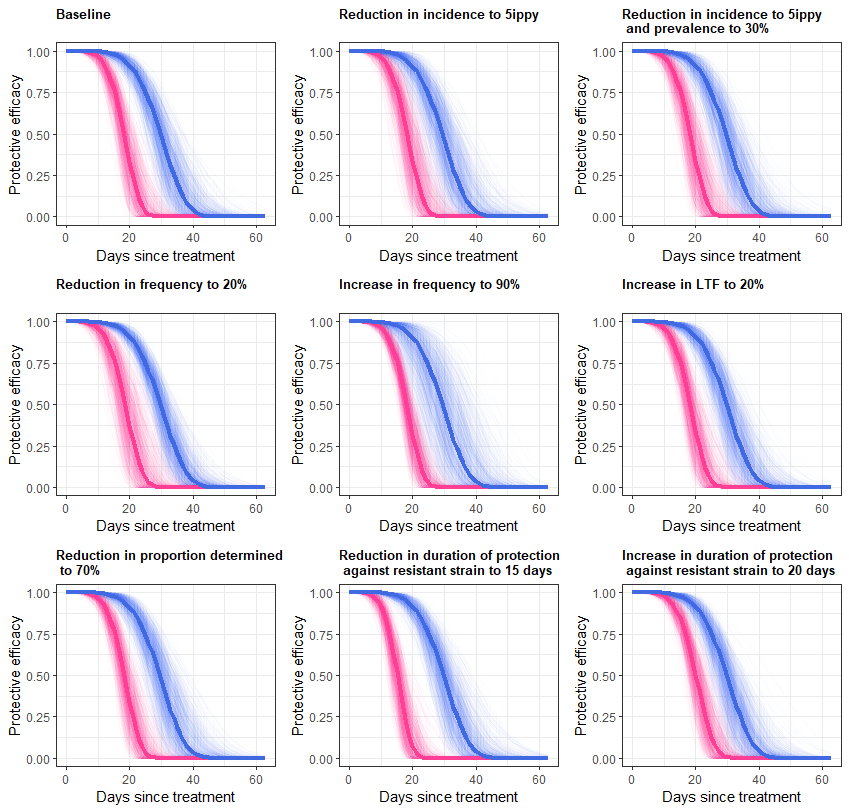


Fig E- Predicted protective efficacy over time against resistant (pink) and sensitive (blue) parasites from time since treatment. Solid line denotes the median of the medians estimated across 1000 simulations, and faint lines show all medians estimated from a 1000 simulations for each scenario related to setting characteristics and effect size.


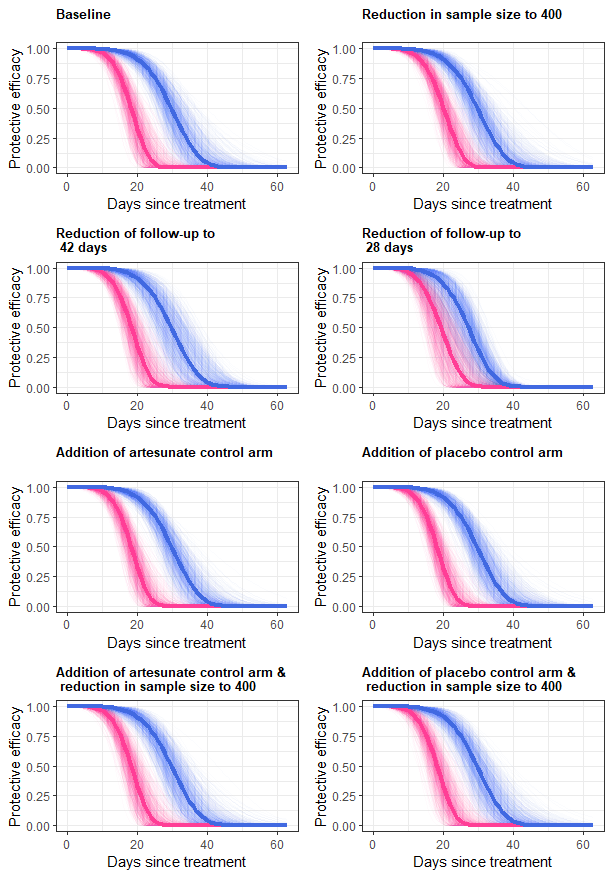


Fig F- Predicted protective efficacy over time against resistant (pink) and sensitive (blue) parasites from time since treatment. Solid line denotes the median of the medians estimated across 1000 simulations, and faint lines show all medians estimated from a 1000 simulations for each scenario related to setting characteristics and effect size.


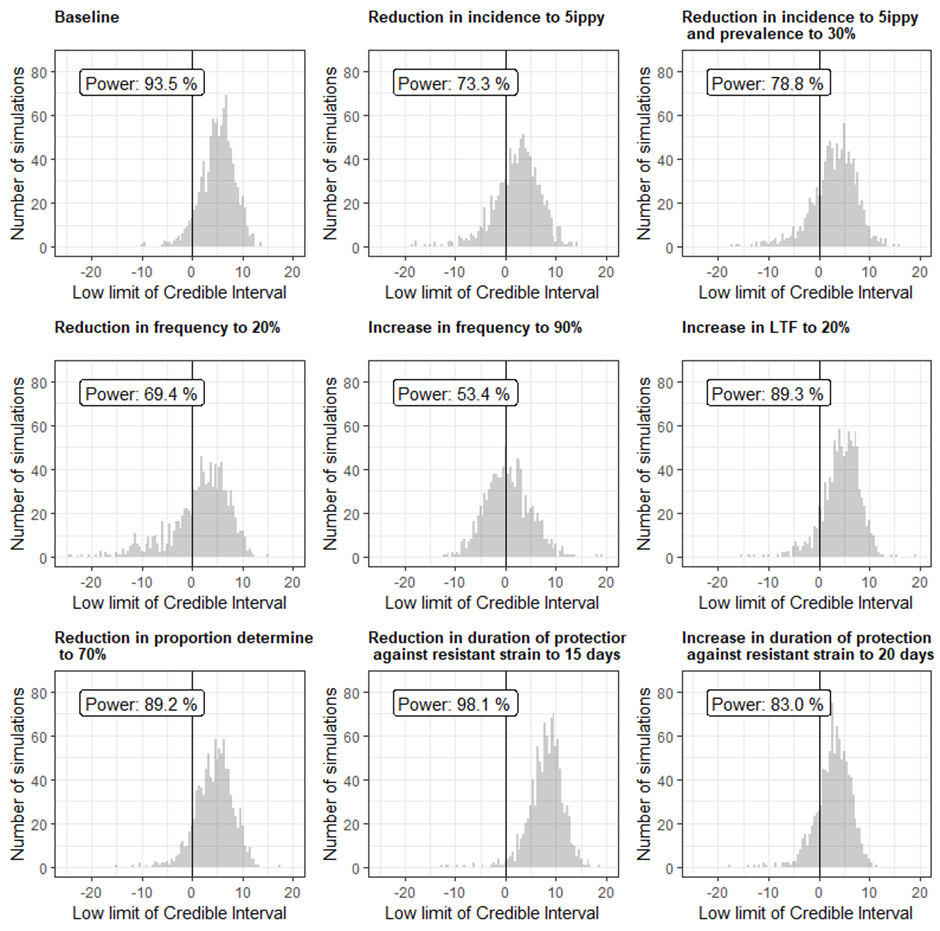


Fig G - Power estimations for detecting a significant difference in the mean duration of protection against two strains (sensitive and resistant) in baseline scenario and scenarios with varying assumptions on setting characteristics and effect size. Histograms display the low limit (2.5^th^ centile) of the 95% Credible Interval estimated for mean difference in protection (mean duration of protection against S minus mean duration of protection against R). Vertical line denotes the value determined by the null hypothesis (difference = 0 days). The magnitude of the values shows variability *within* a single simulation (indicating a wider credible interval), and the spread of the values show variability *between* all simulations.


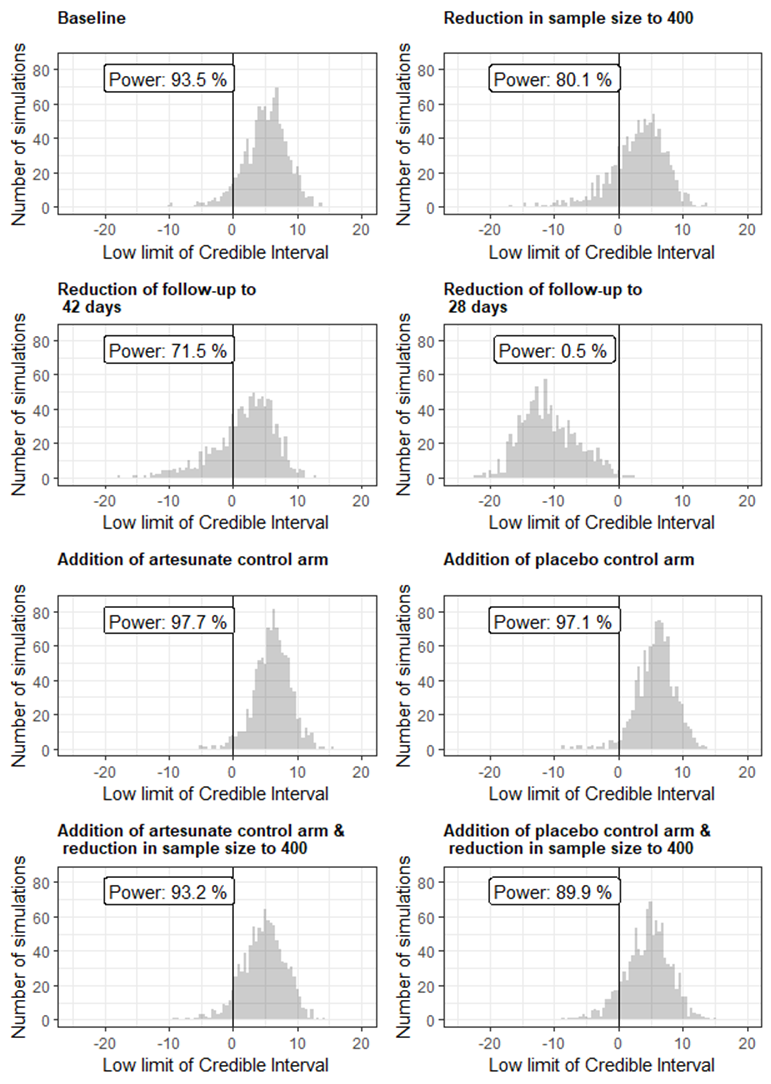


Fig H - Power estimations for detecting a significant difference in the mean duration of protection against two strains (sensitive and resistant) in baseline scenario and scenarios with varying assumptions on study design. Histograms display the low limit (2.5^th^ centile) of the 95% Credible Interval estimated for mean difference in protection (mean duration of protection against S minus mean duration of protection against R). Vertical line denotes the value determined by the null hypothesis (difference = 0 days). The magnitude of the values shows variability *within* a single simulation (indicating a wider credible interval), and the spread of the values show variability in the 2.5^th^ centile *between* all simulations.
