# Supplementary material for: Measuring protective efficacy and quantifying the impact of drug resistance: A novel malaria chemoprevention trial design and methodology
Source: PLoS Med. 2024 May 9;21(5):e1004376. doi: 10.1371/journal.pmed.1004376 (PMC11081503; doi:10.1371/journal.pmed.1004376)
Supplement: S5 File — (DOCX) [file pmed.1004376.s005.docx]

# S5 File - Power calculation using the Cox proportional hazards method


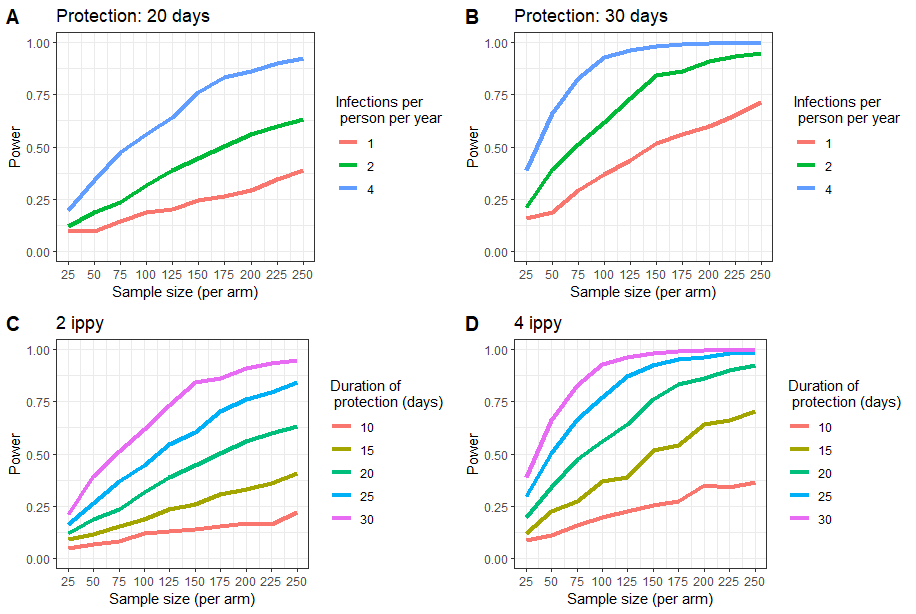


Fig A - Power calculations using a Cox proportional hazards survival method in a two arm controlled-trial shown for different assumptions on duration of protection, transmission level and sample size. The calculations presented are based on 1000 simulations per scenario and the p value of the log likelihood test from the Cox proportional hazards regression. Unlike the main analysis, the scenarios above are simplified and do not include loss to follow-up, or proportion negative on day 0 (assumes that all individuals will be analysed). The scenarios assume the same sample size for both treatment and control arm. The y axis shows the power to detect a difference in time to infection across 63 days of follow-up between treatment and control groups.
