# Supplementary material for: Measuring protective efficacy and quantifying the impact of drug resistance: A novel malaria chemoprevention trial design and methodology
Source: PLoS Med. 2024 May 9;21(5):e1004376. doi: 10.1371/journal.pmed.1004376 (PMC11081503; doi:10.1371/journal.pmed.1004376)
Supplement: S6 File — (DOCX) [file pmed.1004376.s006.docx]

S6 File - Three-strain model
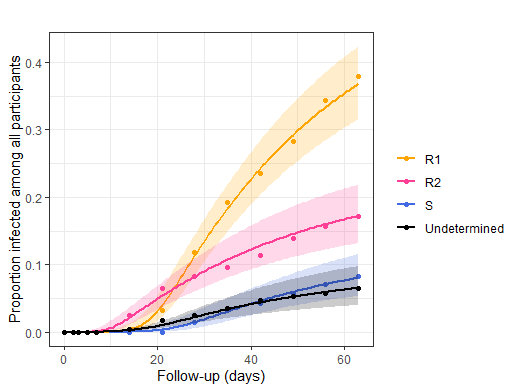


Fig A - An example of a three strain model (lines with 95%CrI ribbons) being fitted to a single simulated dataset (dots) with the following input parameters : sample size=500; frequencies of each strain: R1=70%, R2=6%, S=24%; mean duration of protection against each strain : R1=18 days, R2=10 days, S= 30days.
