# Supplementary material for: Measuring protective efficacy and quantifying the impact of drug resistance: A novel malaria chemoprevention trial design and methodology
Source: PLoS Med. 2024 May 9;21(5):e1004376. doi: 10.1371/journal.pmed.1004376 (PMC11081503; doi:10.1371/journal.pmed.1004376)
Supplement: S7 File — (DOCX) [file pmed.1004376.s007.docx]

# S7 File - Extension of model to include incident mixed infections


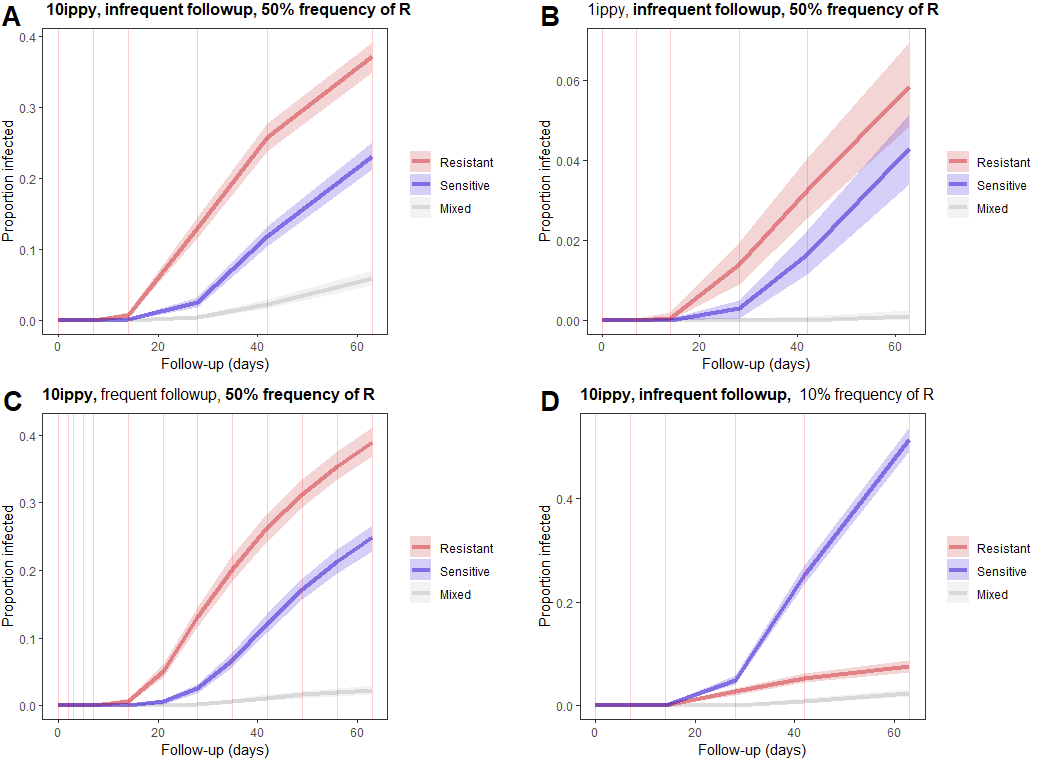


Fig A- The distribution of the proportion of new infections that are with a resistant or sensitive parasite in the simulated data and the proportion of incident mixed infections, across 1000 simulations for each of four scenarios (compared to main analyses, a larger sample size of 2000 individuals was used here to capture any rare ‘incident’ mixed infections). A) high transmission rate, infrequent follow-up, and 90% frequency of resistant (R) strain, B) low transmission rate, infrequent follow-up, and 50% frequency of resistance, C) high infection rate, frequent follow-up and 50% frequency of resistance, D) high infection rate, infrequent follow-up and 10% frequency of resistance. The solid lines denote the median, and shaded areas show the 2.5th and 97.5th percentiles. The vertical red lines indicate the observed time points.

As the analysis includes only time to first infection (rather than the accumulation of infections through follow-up), the occurrence of mixed infections is only an issue when an individual is uninfected and acquires mixed infection by the next observed time-point. We included the probability of an individual being exposed to multiple infections on the same day or on different days, and the probability that each incoming infection is not cleared by the drug. A combination of *all* three factors: high transmission, infrequent follow-up, and relatively equal ratios of resistant-to-sensitive parasites resulted in a low-to-moderate prevalence of ‘incident’ mixed infections during follow-up , but the absence of any of these factors resulted in near-zero ‘incident’ mixed infections.
