# Supplementary material for: Measuring protective efficacy and quantifying the impact of drug resistance: A novel malaria chemoprevention trial design and methodology
Source: PLoS Med. 2024 May 9;21(5):e1004376. doi: 10.1371/journal.pmed.1004376 (PMC11081503; doi:10.1371/journal.pmed.1004376)
Supplement: S8 File — (DOCX) [file pmed.1004376.s008.docx]

# S8 File - Summary table of key messages


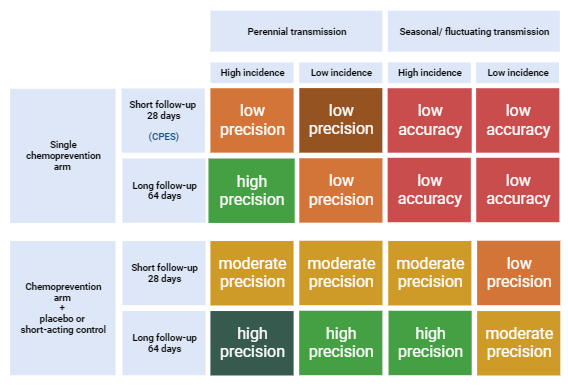


Fig A - Illustrative table showing the ability of a trial to measure efficacy of chemoprevention against *new* infections in settings with different transmission characteristics. The current CPES protocol, marked on the table, is a single chemoprevention arm trial with a short follow-up of 28 days, and defines chemoprevention as the ability to both prevent new infections and clear existing infections. The table is for illustrative purposes as estimated power is dependent on various assumptions (e.g. exact length of follow-up, incidence, expected duration of protection, sample size, etc.).
